# Kynurenine Relaxes Arteries of Normotensive Women and Those With Preeclampsia

**DOI:** 10.1161/CIRCRESAHA.120.317612

**Published:** 2021-03-03

**Authors:** Stephanie A. Worton, Harry A.T. Pritchard, Susan L. Greenwood, Mariam Alakrawi, Alexander E.P. Heazell, Mark Wareing, Adam Greenstein, Jenny E. Myers

**Affiliations:** 1 Maternal & Fetal Health Research Centre, Division of Developmental Biology & Medicine, Faculty of Biology, Medicine and Health, University of Manchester, Manchester, United Kingdom (S.A.W., S.L.G., A.E.P.H., M.W., J.E.M.).; 2 Manchester University Hospital NHS Foundation Trust, Manchester Academic Health Science Centre, Manchester, United Kingdom (S.A.W., A.E.P.H., A.G., J.E.M.).; 3 Division of Cardiovascular Sciences, Faculty of Biology, Medicine and Health, University of Manchester, Manchester, United Kingdom (H.A.T.P., M.A., A.G.).

**Keywords:** hypertension, kynurenine, large-conductance calcium-activated potassium channels, pre-eclampsia, pregnancy, tryptophan

## Abstract

**Rationale:**

Activation of the kynurenine pathway of tryptophan catabolism by infection and inflammation contributes to the development of systemic hypotension. Commercially-available kynurenine has direct vasorelaxant effects on arteries from several species and reduces systemic blood pressure when administered to normotensive or hypertensive rats.

**Objectives:**

To determine whether kynurenine promotes relaxation of human resistance arteries from normotensive and hypertensive pregnant women and to identify the vascular mechanism of its effects.

**Methods and Results:**

In isolated omental and myometrial resistance arteries from normotensive pregnant women, kynurenine (1 mmol/L) significantly reduced U46619-induced constriction (omentum N=14, *P*=2.4×10^−3^; myometrium N=21–25, *P*=2.6×10^−4^) and relaxed preconstricted arteries (N=53, *P*=1.0×10^−11^; N=20, *P*=8.8×10^−3^). Vasorelaxation persisted following endothelium removal (N=7, *P*=1.6×10^−4^) but was completely prevented by inhibition of large-conductance Ca^2+^-activated K^+^ channels (BK_Ca_) channels with iberiotoxin (N=9, *P*=5.7×10^−4^) or paxilline (N=10, *P*=2.1×10^−17^). Accordingly, in isolated vascular smooth muscle cells from omental arteries, kynurenine increased the BK_Ca_ current (n=5–8, *P*=0.022) and the amplitude of spontaneous transient outward currents (n=6, *P*=0.031) but did not affect spontaneous transient outward current frequency. Kynurenine also increased Ca^2+^ spark frequency of pressurized omental arteries (n=8, *P*=0.031). Vasorelaxant effects of kynurenine persisted following inhibition of ryanodine receptors (N=7, *P*=0.48) but were moderately reduced by inhibition of adenylate cyclase (N=9, *P*=0.024). In arteries from women with preeclampsia, kynurenine similarly attenuated vasoconstriction (N=15, *P*=1.3×10^−5^) and induced BK_Ca_-mediated vasodilation (N=16, *P*=2.0×10^−4^). Vasorelaxation in response to kynurenine and a specific BK_Ca_ activator, NS11021, was absent in fetal-derived placental resistance arteries in normal pregnancy and preeclampsia.

**Conclusions:**

Kynurenine dilates systemic arteries from multiple territories via BK_Ca_ activation. Notably, the vasorelaxatory capacity of kynurenine is preserved in preeclampsia, suggesting this approach may have translational potential for the treatment of hypertension in pregnancy. The data warrant further investigation of the potential to exploit this endogenous vasorelaxant as a new treatment for hypertensive pathologies.

## Abstract 2

Supplemental Digital Content is available in the text.

**Meet the First Author, see p 1615**

Kynurenine is an endogenous metabolite derived from tryptophan during the early steps of the kynurenine pathway. In health, kynurenine pathway activity occurs predominantly in the liver under the control of tryptophan 2,3-dioxygenase. However, in response to *Plasmodium* infection or lipopolysaccharide-induced endotoxemia, activity of the inducible enzyme indoleamine 2,3-deoxygenase in endothelium, vascular smooth muscle cells (VSMC), and immune cells,^1–4^ converts tryptophan into vasoactive compounds which contribute to the development of systemic hypotension.^1,5,6^

In vitro, commercially available kynurenine has direct relaxatory effects in arteries from a range of animal species and vascular beds.^1,6–9^ Intravenous kynurenine administration causes an acute reduction in blood pressure (BP) in normotensive (Wistar)^9^ and hypertensive (spontaneously hypertensive)^1^ rats and causes a reduction in pulmonary artery pressure in mouse models of idiopathic pulmonary hypertension.^7^ Furthermore, dietary supplementation with tryptophan-rich grass seed caused a sustained reduction in systolic BP over 60 days, which was largely prevented by inhibiting conversion of tryptophan to kynurenine by indoleamine 2,3-deoxygenase.^10^ These data led to the hypothesis that exogenous kynurenine supplementation could be used to induce therapeutic vasorelaxation in human hypertensive pathologies.

In humans, current evidence of a potential antihypertensive role for the kynurenine pathway is limited to observational data; there is an inverse correlation between kynurenine pathway activity and BP in trauma,^4^ sepsis,^11^ obesity,^12^ and preeclampsia.^13^ To date, evidence of direct vascular effects of kynurenine pathway metabolites on human arteries is limited to a single small study (N=5) in which kynurenine was reported to relax preconstricted omental arteries via activation of type-7 voltage-gated K^+^ channels.^9^

Pregnancy offers an unrivaled opportunity to safely obtain intraabdominal resistance arteries from a healthy, diverse, and motivated population, due to the numerous obstetric indications for laparotomy (cesarean section). This provides a valuable research tool for investigating the effects and mechanisms of vasoactive factors on arteries obtained either from a normotensive population or from the 3% to 5% of women who develop the pregnancy-specific hypertensive condition preeclampsia. Preeclampsia remains a leading global contributor to maternal and offspring morbidity,^14,15^ for which improved vascular treatments are required.

Here, we demonstrate that kynurenine causes relaxation of human resistance arteries sampled from multiple maternal vascular beds and identify the axis of Ca^2+^ spark release from the VSMC sarcoplasmic reticulum to activate large-conductance Ca^2+^-activated K^+^ channels (BK_Ca_) as the mechanism by which kynurenine exerts these effects. Importantly for future translation, the vascular effects of kynurenine persist in arteries from women with preeclampsia.

## Methods

### Data Availability

The data that support the findings of this study are available from the corresponding author upon reasonable request.

Detailed methods can be found in the Data Supplement. In brief, omental, myometrial, or placental biopsies were obtained with informed consent from normotensive women with uncomplicated pregnancies (normal pregnancy) and hypertensive women with a clinical diagnosis of preeclampsia. In isolated resistance arteries mounted on the wire myograph, vasorelaxation was assessed in preconstricted arteries (U46619 80% maximal effective concentration) treated with incremental doses of vehicle or kynurenine (0.05–3 mmol/L) in the presence or absence of established inhibitors of vascular pathways or in mechanically denuded arteries. Constriction in response to U46619 was assessed before and after 1-hour treatment with kynurenine (1 or 6 mmol/L) or vehicle control. Arteries mounted on the pressure myograph were loaded with 4-(6-Acetoxymethoxy-2,7-difluoro-3-oxo-9-xanthenyl)-4′-methyl-2,2′-(ethylenedioxy)dianiline-N,N,N′,N′-tetraacetic acid tetrakis(acetoxymethyl) ester and Ca^2+^ sparks imaged pretreatment and post-treatment with kynurenine (1 mmol/L). Isolated VSMCs were assessed by patch clamp electrophysiology. The whole-cell patch clamp technique was used to assess BK_Ca_ currents following treatment with kynurenine 1 mmol/L or vehicle, and a whole-cell perforated (amphotericin B) patch clamp approach was used to assess spontaneous transient outward currents (STOCs) in VSMCs in the absence or presence of kynurenine (1 mmol/L).

## Results

The characteristics of 164 healthy, normotensive pregnant women and 35 women with preeclampsia included in this study are shown in the Table. By definition, women with preeclampsia had multiple BP recordings ≥140/90 mm Hg, and all of these women received antihypertensive treatment in the antenatal period.

**Table.**
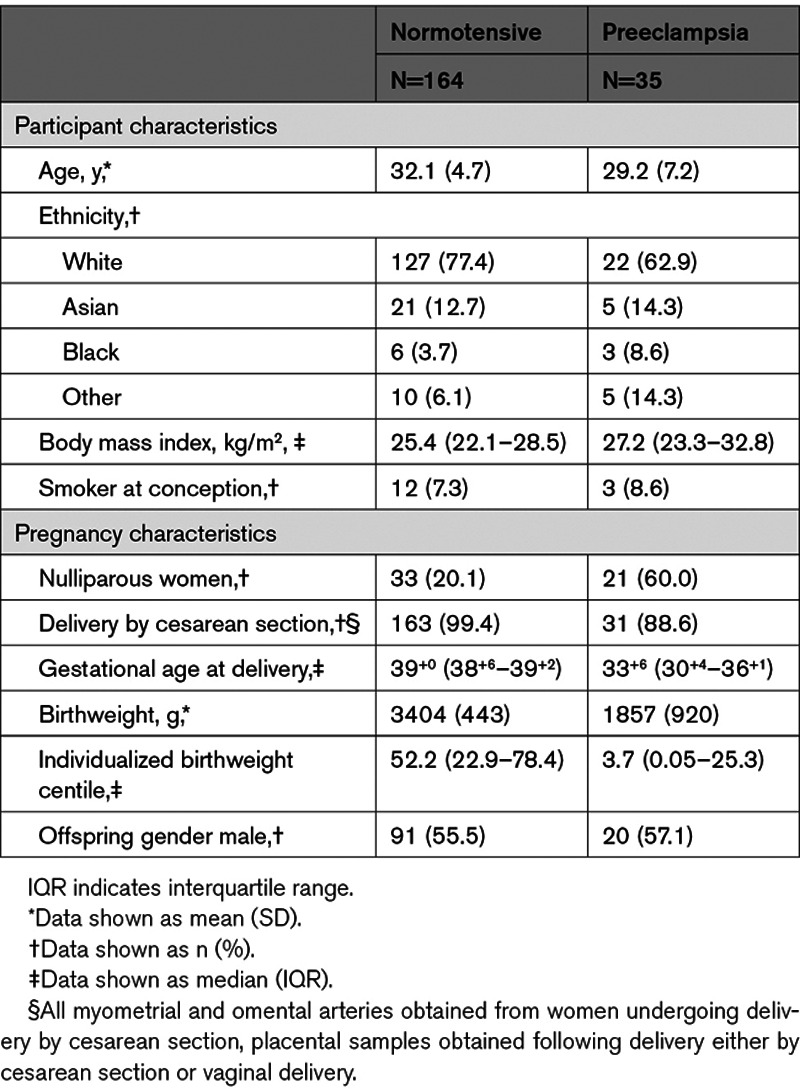
Characteristics of Normotensive and Preeclamptic Participants

### Kynurenine Causes Relaxation and Reduces U46619-Induced Constriction in Omental and Myometrial Arteries From Normotensive Pregnant Women

**Figure 1.**
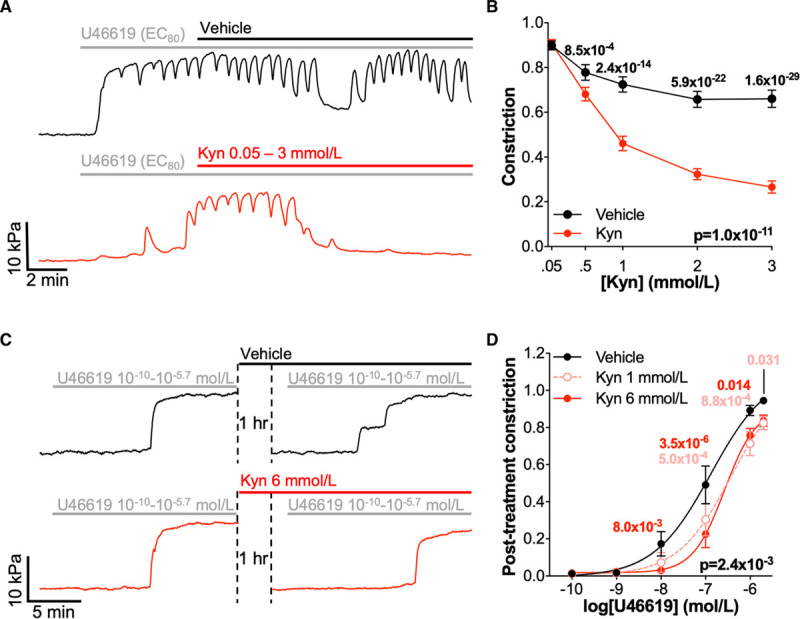
**Kynurenine (Kyn) causes vasorelaxation and reduces vasoconstriction in omental arteries.** **A**, Representative myography traces of preconstricted omental arteries from the same woman treated with increasing doses of vehicle (physiological saline solution [PSS]; black) or Kyn (0.05–3 mmol/L; red). **B**, Cumulative post-treatment concentration-relaxation curves for preconstricted omental arteries (n=85–91, N=53). Kyn- and vehicle-treated arteries compared by 2-way repeated-measures (RM) ANOVA with Sidak multiple comparison tests. Data are not normally distributed. **C**, Representative myography traces of individual omental arteries constricted with increasing doses of U46619 (10^-10^–10^-5.7^ mol/L) before and after 1 h treatment with vehicle (PSS; black) or Kyn 6 mmol/L (red). **D**, Cumulative concentration-response curves for U46619 (10^-10^–10^-5.7^ mol/L) following 1 h treatment with Kyn 1 mmol/L or 6 mmol/L or vehicle control (N=14). Curves compared by 2-way RM ANOVA with Dunnett multiple comparison to control-treated arteries. Data are not normally distributed. EC_80_ indicates 80% maximal effective concentration.

Kynurenine induced relaxation of submaximally constricted (80% maximal effective concentration) arteries isolated from the omentum of normotensive pregnant women (n=85–91, N=52; diameter 313±64 μm; Figure 1A and 1B). Oscillations in tone were apparent, which are commonly observed in submaximally constricted maternal arteries.^16^ After 1 hour, constriction of omental arteries was reduced on repeated exposure to U46619, even in control-treated arteries (pretreatment versus post-treatment constriction; N=15; 2-way ANOVA, *P*=0.038). However, treatment for 1 hour with kynurenine 1 or 6 mmol/L significantly reduced subsequent U46619-induced constriction, compared with vehicle control (N=15; diameter 301±60 µm; Figure 1C and 1D). Treatment with kynurenine 6 mmol/L significantly increased the concentration of U46619 required to cause 50% maximal constriction (EC_50_), indicating a reduction in sensitivity (change in −logEC_50_ [ΔpEC_50_] control −0.248 [−0.625 to −0.022] versus kynurenine 6 mmol/L −0.822 [−0.992 to −0.476]; N=12; Wilcoxon signed-rank test, *P*=0.012).

Assessments of the effects of kynurenine on preconstricted arteries and U46619-induced constriction were repeated in arteries isolated from the myometrium, a maternal vascular bed which undergoes substantial remodeling and receives prioritized perfusion in pregnancy (Figure I in the Data Supplement). As in omental arteries, submaximally constricted myometrial arteries demonstrated significant relaxation in response to kynurenine (n=26–29, N=20; diameter 289±91 μm; Figure IA and IB in the Data Supplement). Upon repeated exposure to U46619, the reduction in constriction observed for omental arteries was exaggerated in control-treated myometrial arteries (pretreatment versus post-treatment constriction, N=25; 2-way ANOVA, *P*=4.0×10^−8^). Treatment of myometrial arteries with kynurenine further attenuated constriction in response to U46619 (N=25; diameter 280±94 µm; Figure IC and ID in the Data Supplement). Treatment with 6 mmol/L kynurenine caused a significantly larger rightward shift in pEC_50_ (ΔpEC_50_ control, −0.151 [−0.487–0.064] versus kynurenine, 6 mmol/l −0.553 [−0.763 to −0.248]; N=17; Wilcoxon signed-rank tests, *P*=0.017).

In either omental or myometrial arteries, exposure to kynurenine did not affect basal tone of relaxed arteries or subsequent maximal constriction in response to high-potassium physiological saline solution.

### Kynurenine Causes Endothelium-Independent Relaxation via Activation of BK_Ca_ Channels

**Figure 2.**
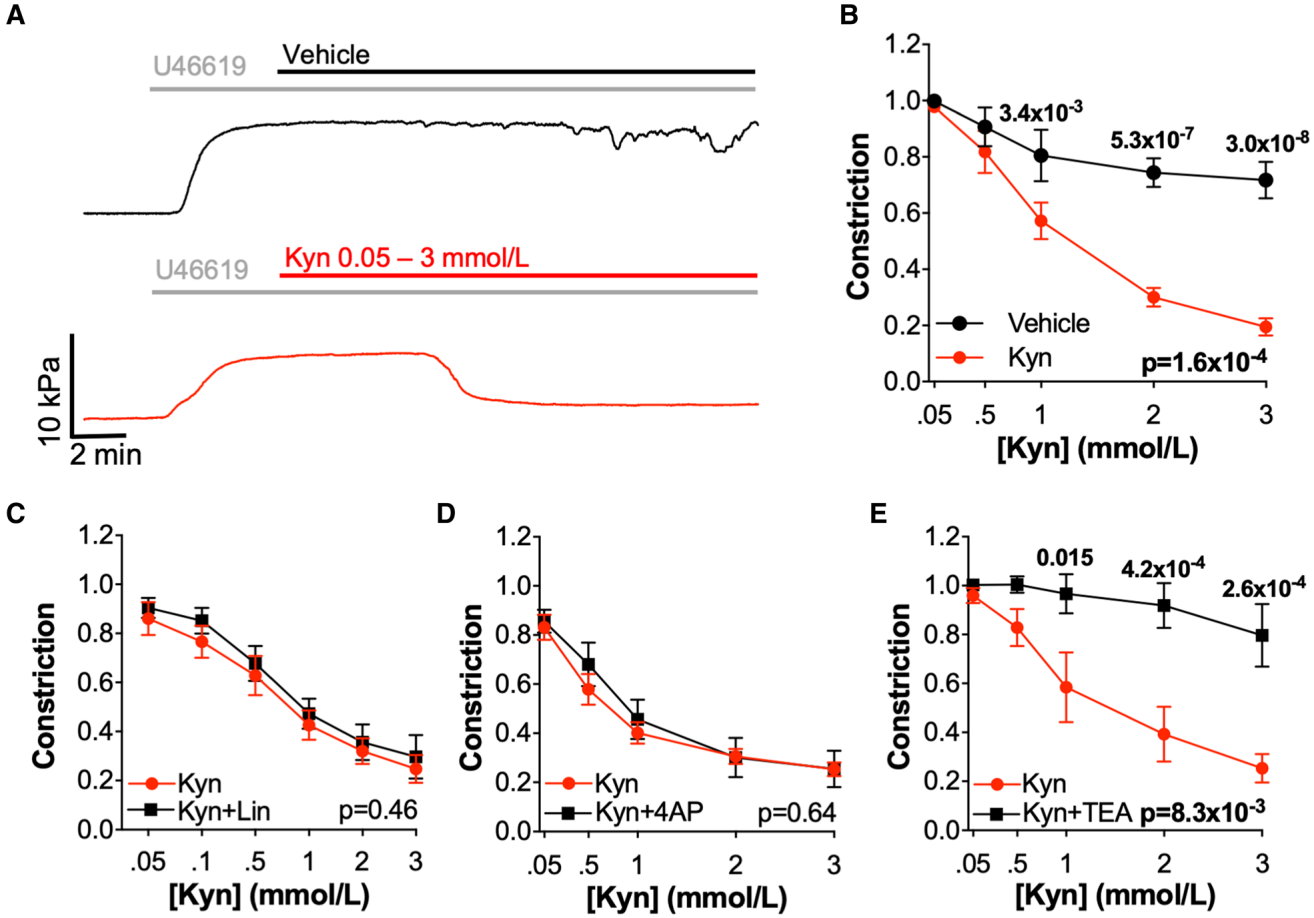
**Kynurenine (Kyn) causes endothelium-independent relaxation via a K^+^ channel other than type-7 K_v_ channels.** **A**, Representative myography traces of preconstricted denuded omental arteries from the same woman treated with increasing doses of vehicle (physiological saline solution; black) or Kyn (0.05–3 mmol/L; red). **B**, Cumulative concentration-response curves for preconstricted denuded omental arteries treated with Kyn (0.05–3 mmol/L) or vehicle (n=10–11, N=7). **C–E**, Endothelium intact omental arteries treated±inhibitors before preconstriction (U46619 80% maximal effective concentration) and addition of incremental concentrations of Kyn (0.05–3 mmol/L). Pretreatment with inhibitors (black) or vehicle control (red) as follows; (**C**) linopirdine 10 μmol/L (Lin; type-7 K_v_ inhibitor; n=11–12, N=7), (**D**) 4-aminopyridine 1 mmol/L (4AP; K_v_ inhibitor; n=9–10, N=6), (**E**) Tetraethylammonium chloride 5 mmol/L (TEA; nonspecific K^+^ channel inhibitor; n=10–13, N=6), **B–E**, curves compared by 2-way repeated-measures ANOVA with Sidak multiple comparison tests.

Having established a consistent and substantial effect of kynurenine on human resistance arteries, the mechanism through which kynurenine induces vasorelaxation was investigated using omental arteries obtained from healthy, normotensive pregnant women. Vasorelaxation in response to kynurenine persisted in omental arteries in which the endothelium had been mechanically removed (Figure 2A and 2B), indicating an endothelium-independent mechanism of vascular relaxation. Removal of the endothelium, which abolished vasomotion, was confirmed by absent relaxation in response to bradykinin (endothelium intact: n=167, N=45, remaining constriction 0.349 [0.146–0.806] versus denuded arteries, n=21, N=7, 0.927 [0.896–0.978]).

Inhibition of type-7 K_v_ channels has been reported to prevent kynurenine-induced relaxation in human omental and rat mesenteric arteries.^9^ However, in omental arteries from normotensive pregnant women, there was no change in kynurenine-induced relaxation following pretreatment with a specific type-7 K_v_ channel inhibitor, linopirdine 10 μmol/L (Figure 2C), or a nonspecific K_v_ family inhibitor 4-aminopyridine 1 mmol/L (Figure 2D). All inhibitors that had no effect on kynurenine-induced relaxation were confirmed to act upon their target pathways within this experimental model (Figure II in the Data Supplement). Tetraethylammonium chloride 5 mmol/L, a nonspecific inhibitor of several K^+^ channel subtypes, completely prevented all relaxation in response to kynurenine (Figure 2E). Abolition of kynurenine-induced relaxation in the presence of tetraethylammonium chloride, but not linopirdine or 4-aminopyridine, indicated the involvement of a K^+^ channel outwith the K_v_ family.

**Figure 3.**
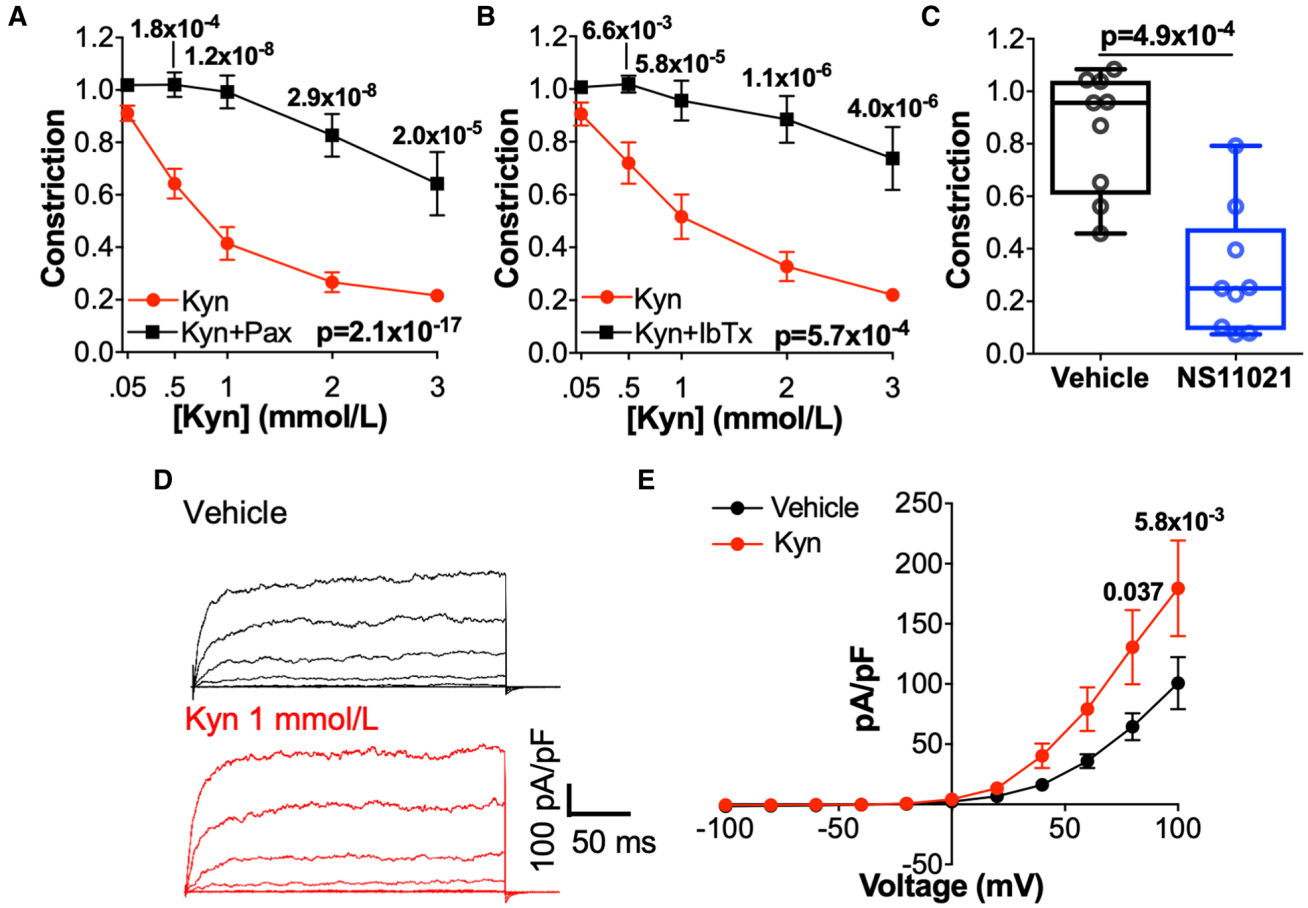
**Kynurenine (Kyn) causes vasorelaxation via large-conductance Ca2+-activated K+ channels (BK_Ca_) channels.** **A** and **B**, Relaxation of preconstricted omental arteries in response to Kyn (0.05–3 mmol/L) following pretreatment with vehicle control (physiological saline solution [PSS]) or BK_Ca_ inhibitors (**A**) paxilline 10 μmol/L (Pax; n=15–20, N=10) or (**B**) Iberiotoxin 100 nmol/L (IbTx; n=16–17, N=9). **C**, Preconstricted omental arteries treated with NS11021 10 μmol/L or vehicle control (0.1% ethanol; n=15, N=9). Remaining constriction compared by Wilcoxon signed-rank test. **D** and **E**, Paxilline-sensitive BK_Ca_ currents in isolated vascular smooth muscle cells (VSMCs) recorded using the ruptured whole-cell configuration of the patch clamp technique (−100 to +100 mV) treated with vehicle (dispersal PSS) or Kyn 1 mmol/L. **D**, Representative currents recorded in response to voltage-steps in individual VSMCs. **E**, Paxilline-sensitive (BK_Ca_) current-voltage curves (n=5–8, N=4). **A**, **B**, and **E**, Data are not normally distributed. Curves compared by 2-way repeated-measures ANOVA with Sidak multiple comparison tests.

We next hypothesized that kynurenine acts via BK_Ca_. Following treatment of arteries with highly specific inhibitors of BK_Ca_ - paxilline 10 μmol/L (Figure 3A) or iberiotoxin 100 nmol/L (Figure 3B) - relaxation in response to kynurenine was completely abolished. Arteries treated with BK_Ca_ inhibitors relaxed in response to the endothelium-dependent vasodilator bradykinin, and following washout, there was no difference in their constriction to high-potassium physiological saline solution. The specific BK_Ca_ activator NS11021 10 μmol/L caused significant relaxation of preconstricted omental arteries (Figure 3C), which was of similar magnitude to that caused by kynurenine. Whole-cell patch clamp electrophysiology confirmed that the paxilline-sensitive current, mediated by BK_Ca_, was significantly increased by kynurenine 1 mmol/L (Figure 3D and 3E). Meanwhile, the small, residual paxilline-insensitive outward current was reduced by kynurenine (Figure III in the Data Supplement), providing further confidence that kynurenine does not induce vasodilation via activation of K_v_ channels.

**Figure 4.**
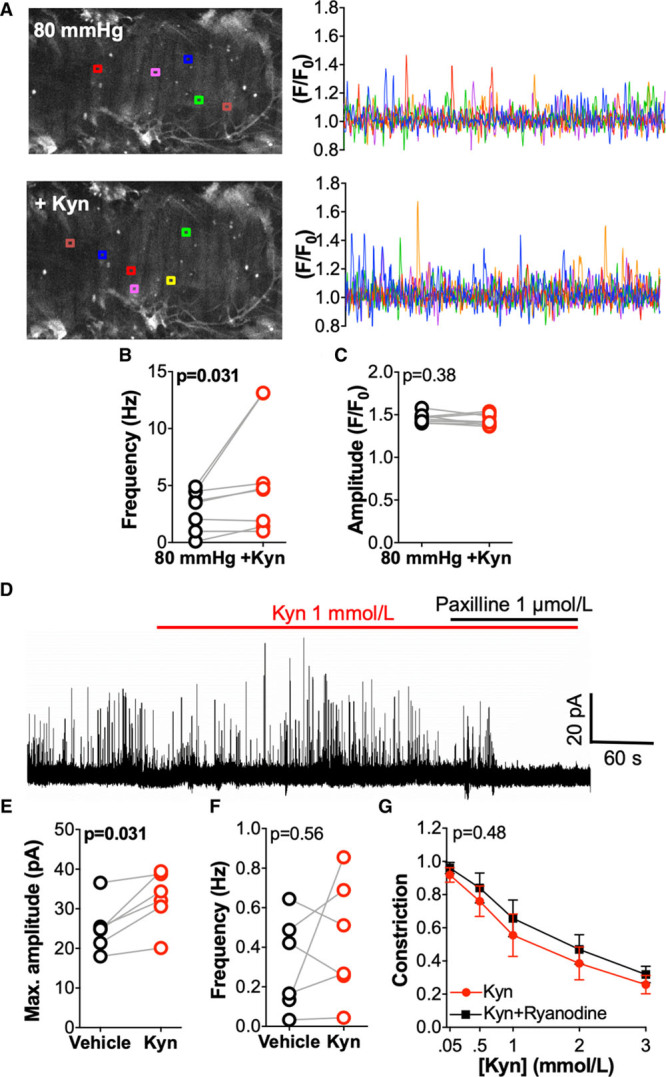
**Kynurenine (Kyn) increases vascular smooth muscle cell (VSMC) Ca^2+^ sparks but does not affect spontaneous transient outward current (STOC) frequency.** **A–C**, Ca^2+^ spark activity in omental arteries pressurized at 80 mm Hg. **A**, Representative data from a single artery at baseline (80 mm Hg; **top**) and following Kyn 1 mmol/L (+Kyn; **bottom**). Colored lines (**right**) indicate changes in fractional fluorescence (F/F_0_) at individual regions corresponding to boxes shown in the artery image (**left**). **B**, Cumulative Ca^2+^ spark frequency (n=8, N=5). **C**, Cumulative Ca^2+^ spark amplitude (n=8, N=5). **D–F**, STOCs (recorded at −20 mV) measured at baseline (control) and after treatment with Kyn 1 mmol/L, in VSMCs isolated from omental arteries and studied using the perforated patch configuration of the whole-cell patch clamp technique. **D**, Representative recording of STOCs in VSMCs, which were sensitive to the large-conductance Ca^2+^-activated K^+^ channels channel inhibitor paxilline. **E**, Cumulative STOC amplitude (n=6, N=4). **F**, Cumulative STOC frequency (n=6, N=4). **G**, Relaxation of preconstricted (U46619 80% maximal effective concentration) omental arteries in response to Kyn (0.05–3 mmol/L) following pretreatment with vehicle control (physiological saline solution) or ryanodine 20 μmol/L (ryanodine receptor inhibitor; n=13–14, N=7). Compared by 2-way repeated-measures ANOVA. **B**, **C**, **E**, and **F**, Compared by Wilcoxon signed-rank test.

The physiological source of Ca^2+^ for the activation of BK_Ca_ channels in VSMCs is Ca^2+^ release events from ryanodine receptors (Ca^2+^ sparks) on the sarcoplasmic reticulum membrane.^17^ To determine if kynurenine-induced BK_Ca_-mediated dilation is via an increase in Ca^2+^ spark activity, we recorded Ca^2+^ sparks from 4-(6-Acetoxymethoxy-2,7-difluoro-3-oxo-9-xanthenyl)-4′-methyl-2,2′-(ethylenedioxy)dianiline-N,N,N′,N′-tetraacetic acid tetrakis(acetoxymethyl) ester loaded, pressurized omental arteries (Figure 4A). Application of kynurenine (1 mmol/L) significantly increased the frequency of Ca^2+^ sparks, albeit to a variable degree, without affecting the amplitude (Figure 4B and 4C). To determine if the increase in Ca^2+^ sparks by kynurenine augmented BK_Ca_ activity, we performed perforated patch experiments in isolated omental VSMCs to record STOCs, a measurement of the transient activation of BK_Ca_ channel by Ca^2+^ sparks (Figure 4D). Interestingly, we found that kynurenine (1 mmol/L) increased STOC amplitude but not frequency (Figure 4E and 4F). Consistent with this observation, kynurenine-induced relaxation of omental arteries was not affected by inhibition of ryanodine receptors with ryanodine 20 μmol/L (Figure 4G).

**Figure 5.**
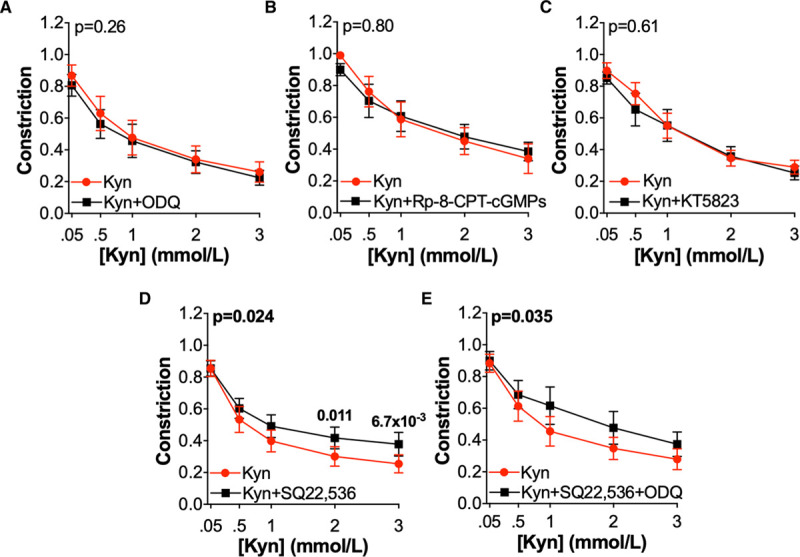
**The role of the adenylate cyclase and guanylate cyclase pathways in kynurenine (Kyn)-induced relaxation.** Preconstricted omental arteries were treated with incremental concentrations of Kyn (0.05–3 mmol/L) following treatment with inhibitors (black) or vehicle control (red) as follows; **A**, 1H-[1,2,4]oxadiazolo[4,3,-a]quinoxaline-1–one (ODQ) 10 μmol/L (oxidizes sGC to Fe^3+^ form; n=10–11, N=7) **B**, 8-(4-Chlorophenylthio)-guanosine 3′,5′-cyclic monophosphorothioate (Rp-8-CPT-cGMPs) 1 mmol/L (inhibits cGMP activation of PKG [protein kinase G]; n=11–13, N=6). **C**, KT5823 1 μmol/L (ATP-competitive inhibition of PKG; n=8–10, N=6). **D**, 9-(tetrahydro-2-furanyl)-9H-purin-6-amine (SQ22,536) 100 μmol/L (adenylate cyclase inhibitor; n=12–14, N=9), **E**, SQ22,536 100 μmol/L + ODQ 10 μmol/L (n=13–14, N=9). Curves compared by 2-way repeated-measures ANOVA with Sidak multiple comparison tests.

Activation of both the GC (guanylate cyclase) and AC (adenylate cyclase) pathways has been implicated in partially mediating kynurenine-induced relaxation in porcine coronary arteries,^1^ and these pathways are known regulators of BK_Ca_ activity. In omental arteries from pregnant women, kynurenine-induced relaxation was unaffected by treatment with 1H-[1,2,4]oxadiazolo[4,3,-a]quinoxaline-1–one (Figure 5A), which oxidizes NO-sensitive Fe^2+^-containing GC to its nitric oxide-insensitive Fe^3+^/heme-free form. As kynurenine has previously been shown to activate both Fe^2+^ and Fe^3+^ forms of GC,^1^ downstream pathway inhibitors were also used; neither inhibition of cGMP activation of PKG (protein kinase G) with 8-(4-Chlorophenylthio)-guanosine 3′,5′-cyclic monophosphorothioate (Figure 5C) nor ATP-competitive inhibition of PKG with KT5823 (Figure 5C) affected kynurenine-induced relaxation. The efficacy of these inhibitors to reduce GC pathway-mediated relaxation by sodium nitroprusside was confirmed (Figure IID through IIF in the Data Supplement).

Pretreatment of omental arteries with the AC inhibitor 9-(tetrahydro-2-furanyl)-9H-purin-6-amine (SQ22,536) did cause a small, but statistically significant, attenuation in relaxation in response to kynurenine (Figure 5D). However, in SQ22,536-treated arteries, relaxation in response to kynurenine remained significantly greater than control-treated arteries (final constriction vehicle only 0.886±0.087 versus kynurenine+SQ22,536 0.356±0.098; n=14, N=7; 2-way repeated-measures [RM] ANOVA, *P*=0.041). This effect of SQ22,536 was consistently observed, although without any additive effect when SQ22,536 was used in combination with 1H-[1,2,4]oxadiazolo[4,3,-a]quinoxaline-1–one (Figure 5E).

### Effects of Kynurenine Are Preserved in Omental Arteries From a Hypertensive Cohort

**Figure 6.**
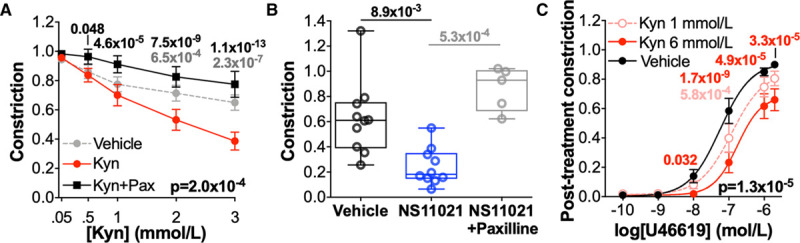
**Kynurenine (Kyn) induces large-conductance Ca2+-activated K+ channels–mediated relaxation and attenuates vasoconstriction in omental arteries from a hypertensive cohort.** **A**, Omental arteries from women with preeclampsia treated±paxilline 10 μmol/L (Pax) were preconstricted (80% maximal effective concentration) and exposed to increasing concentrations of Kyn (0.05–3 mmol/L) or vehicle (physiological saline solution; n=28–29, N=16). Curves compared by 2-way repeated-measures (RM) ANOVA with Dunnett multiple comparisons to Kyn-treated arteries. **B**, Preconstricted omental arteries from women with preeclampsia treated with vehicle (0.1% ethanol; n=8, N=10) or NS11021 10 μmol/L (n=10, N=10) or NS11021 10 μmol/L+paxilline (n=6, N=5). Remaining constriction compared by Kruskal-Wallis test with Dunn multiple comparisons to NS11021-treated arteries. **C**, Constriction of omental arteries from women with preeclampsia in response to U46619 (10^-10^–10^-5.7^ mol/L) following 1 h treatment with Kyn 1 mmol/L or 6 mmol/L or vehicle control (N=15). Curves compared by 2-way RM ANOVA with Dunnett multiple comparison tests. **A–C**, Data are not normally distributed.

To assess the translational potential of kynurenine to treat hypertension, the vasorelaxatory capacity of kynurenine was assessed in arteries from a hypertensive cohort (women with preeclampsia). Kynurenine relaxed preconstricted omental arteries (diameter 296±73 μm) from women with preeclampsia, which was prevented by pretreatment with the BK_Ca_ inhibitor paxilline (Figure 6A). Omental arteries from women with preeclampsia also relaxed substantially in response to the BK_Ca_ activator NS11021 10 μmol/L (Figure 6B).

Treatment of omental arteries from women with preeclampsia with kynurenine 1 or 6 mmol/L caused dose-dependent attenuation of subsequent constriction in response to U46619 compared with control-treated arteries (Figure 6C). A significant rightward shift in ΔpEC_50_ occurred following treatment with kynurenine 6 mmol/L (control, −0.300 [−0.508 to −0.061] versus kynurenine 6 mmol/L −0.734 [−0.554 to −0.846]; N=14, Wilcoxon signed-rank test *P*=0.027).

In a direct comparison of arteries from unmatched normotensive and preeclamptic women, there were no baseline differences in arterial diameter or U46619 pEC_50_, but arteries from women with preeclampsia had a significant increase in baseline maximal constriction in response to both high-potassium physiological saline solution (normotensive, N=39, 11.75±0.336 kPa versus preeclampsia, N=18, 12.23±0.374 kPa; unpaired *T* test, *P*=0.032) and U46619 (13.23±0.359 kPa versus 14.99±0.438 kPa; N=18 or 39, unpaired *T* test, *P*=2.0×10^−3^). There was no significant difference in the spontaneous relaxation occurring in vehicle-treated arteries from women with preeclampsia or normotensive women (preeclampsia data shown in Figure 6A versus normotensive data shown in Figure 1B; 2-way RM ANOVA, *P*=0.47). However, omental artery relaxation in response to kynurenine was reduced in women with preeclampsia when directly compared with women with normal pregnancy (2-way ANOVA, *P*=4.6×10^−3^). The greatest difference in relaxation was observed at 1 mmol/L kynurenine (remaining constriction preeclampsia 0.698±0.068 versus normotensive 0.471±0.048; Sidak postcomparison test, *P*=1.7×10^−3^), but final relaxation at the maximal dose was not significantly different (remaining constriction preeclampsia 0.378±0.058 versus normotensive 0.244±0.023; Sidak postcomparison test, *P*=0.16). There was no significant difference in final relaxation in response to NS11021 between normotensive women and those with preeclampsia (preeclampsia data shown in Figure 6B versus normotensive data shown in Figure 3C; 2-way RM ANOVA, *P*=0.14).

The effects of kynurenine treatment on constriction in response to U46619 were comparable between normotensive women and women with preeclampsia; following treatment with kynurenine 1 mmol/L or 6 mmol/L, the change in maximal constriction and ΔpEC_50_ were equivalent between women with normal pregnancy (N=15/group) and those with preeclampsia (N=12/group; Mann-Whitney tests, all *P*>0.05).

### Kynurenine Reduces U46619-Induced Vasoconstriction but Does Not Cause Vasodilation in Placental Arteries

**Figure 7.**
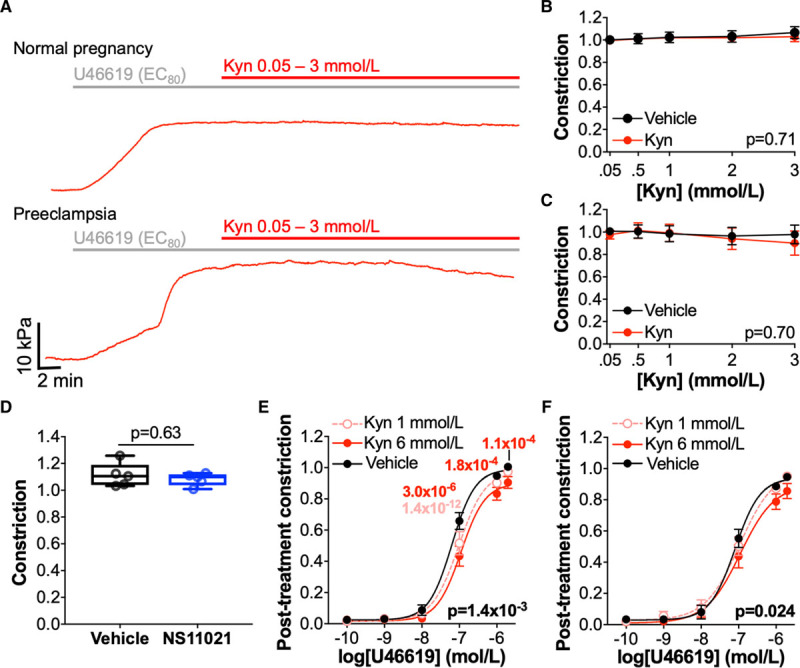
**Kynurenine (Kyn) does not cause relaxation in placental arteries but does reduce constriction.** **A**, Representative myography traces of individual placental chorionic plate arteries from women with normal pregnancy (**top**) or preeclampsia (**bottom**) preconstricted (80% maximal effective concentration [EC_80_]) with U46619 and treated with incremental doses of Kyn (0.05–3 mmol/L). **B** and **C**, Cumulative concentration-response curves to Kyn (0.05–3 mmol/L) or vehicle (physiological saline solution [PSS]) for preconstricted placental arteries obtained from women with (**B**) normal pregnancy (n=14–15, N=10), or (**C**) preeclampsia (n=19–20, N=12). Curves compared by 2-way repeated-measures (RM) ANOVA. **D**, Preconstricted placental arteries from normotensive pregnant women treated with NS11021 10 μmol/L or vehicle control (0.1% ethanol; n=9–10, N=5). Remaining constriction compared by Wilcoxon signed-rank test. **E** and **F**, Concentration-response curves for U46619 (10^-10^–10^-5.7^ mol/L) following 1 h treatment with Kyn 1 mmol/L or 6 mmol/L or vehicle (PSS) in placental arteries from women with (**E**) normal pregnancy (N=15) or, (**F**) preeclampsia (N=14). Curves compared by 2-way RM ANOVA with Dunnett multiple comparison tests. **C**, **E**, and **F**, Data are not normally distributed.

A prospective antihypertensive for use in pregnancy would ideally reduce maternal BP without compromising the fetal circulation. As a first step in determining the potential fetal hemodynamic effects of using kynurenine to treat hypertension in pregnancy, we assessed the effects of kynurenine on the fetal-placental circulation using fetally-derived, resistance arteries from the placenta, which receives approximately one-third of total fetal cardiac output in utero. Kynurenine ≤3 mmol/L did not cause relaxation in preconstricted placental arteries from either normotensive women (diameter, 308±125 μm; N=10; Figure 7A and 7B) or women with preeclampsia (282±138 μm; N=12; Figure 7A and 7C). In placental arteries from normotensive pregnancies, the lack of relaxation in response to BK_Ca_-activating kynurenine was confirmed by the absence of relaxation in response to the BK_Ca_ activator NS11021 (Figure 7D).

Treatment with kynurenine for 1 hour attenuated subsequent U46619-induced constriction in placental arteries, from women with normotensive pregnancy (Figure 7E) and women with preeclampsia (Figure 7F), but effects were smaller than those seen in maternal arteries. In placental arteries from normotensive women, treatment with kynurenine 6 mmol/L caused a rightward shift in pEC_50_ (ΔpEC_50_ control, 0.015 [−0.026 to 0.070] versus kynurenine, 6 mmol/L −0.194 [−0.671 to −0.044]; N=13; Wilcoxon signed-rank test, *P*=2.4×10^−3^). However, in placental arteries from women with preeclampsia, ΔpEC_50_ was not significantly different between control and kynurenine-treated arteries (ΔpEC_50_ control, −0.130 [−0.190 to −0.047] versus kynurenine, 6 mmol/L −0.156 [−0.429 to 0.059]).

Placental arteries from women with normotensive pregnancy and women with preeclampsia were of comparable diameter and baseline U46619 pEC_50_, but placental vessels from women with preeclampsia demonstrated greater maximal constriction in response to U46619 and high-potassium physiological saline solution compared with arteries from normotensive women. The apparent reduction in effect of kynurenine pretreatment on arteries from women with preeclampsia appears to be due to a greater attenuation in constriction in control-treated arteries on repeated exposure to U46619 (control-treated arteries from normal pregnancy shown in Figure 7E versus control-treated arteries from preeclampsia shown in Figure 7F; 2-way RM ANOVA; *P*=0.026). This may represent a washout of the preeclampsia phenotype over time. Compared with normotensive pregnancy (N=15), control-treated placental arteries from women with preeclampsia (N=14) had a larger reduction in maximal constriction (Mann-Whitney test, *P*=0.054 [trend]) and ΔpEC_50_ (Mann-Whitney test, *P*=0.020) on their second exposure to U46619, whereas the change in maximal constriction and ΔpEC_50_ after treatment with kynurenine 1 mmol/L or 6 mmol/L was comparable between arteries from women with normal pregnancy (N=15) and preeclampsia (N=14; Mann-Whitney tests, all *P* values >0.05).

## Discussion

Data presented here represent the most comprehensive study of the effects of kynurenine on human resistance arteries to date. The prorelaxant effect of kynurenine is confirmed in human arteries and our study provides further translational impetus as we have included a well-characterized cohort of women with preeclampsia. There are 4 novel findings from our study: First, kynurenine-induced relaxation occurs in arteries from women with preeclampsia to a degree that suggests potential for clinical application. Second, we have identified the Ca^2+^ spark-to-BK_Ca_ channel vasoregulatory axis as the mechanism by which kynurenine induces arterial relaxation. Third, we have shown the vasorelaxant potential of BK_Ca_ activators (kynurenine and NS11021) is intact in women with preeclampsia. Finally, the vasorelaxant effects of both kynurenine and NS11021 are absent in placental chorionic plate arteries.

Hypertension in preeclampsia is driven by widespread endothelial dysfunction, increased sensitivity to vasoconstrictors, and impaired endothelium-dependent relaxation. Despite advances in medical care, delivery of the fetus and placenta remains the only cure for preeclampsia. Current antihypertensive strategies are often problematic and severe maternal hypertension due to its association with cerebrovascular accident, remains the leading indicator for iatrogenic preterm delivery in preeclampsia^18^; improving the management of hypertension is an important clinical target to minimize maternal complications and safely allow delivery to be delayed.

The kynurenine pathway has been increasingly investigated over the last decade due to the vasorelaxant, immunoregulatory, and antioxidant properties of pathway metabolites. We have postulated that these properties may provide a multi-faceted approach to treat several pathophysiological features of preeclampsia, particularly those associated with microvascular dysfunction.^19^ Here, we have built upon previous limited evidence of the vascular effect of kynurenine in human arteries^9^: using resistance arteries from 98 normotensive pregnant women, we have demonstrated a large, reproducible prorelaxant effect of kynurenine in 2 maternal vascular beds. Direct vasorelaxatory responses were similar to those reported by Wang et al^1^ using porcine coronary artery and mouse aorta. In previous studies, the kynurenine concentration required for vasorelaxation of preconstricted arteries ranged from 0.03 to 9 mmol/L^7–9^; this wide range of effective concentrations might be attributable to methodological differences or to variation in sensitivity between species and vascular beds. Treatment of human resistance arteries with kynurenine 1 mmol/L for 1 hour reduced maximal constriction and sensitivity to a thromboxane-mimetic vasoconstrictor; these effects are concordant with previous observations in porcine coronary artery^1^ and rat aorta,^8^ although notably, we have demonstrated effects at a lower concentration than previously tested. Although Watts et al^8^ postulated that vasorelaxant effects attributed to kynurenine pathway metabolites could be explained by the effects of solvents or pH, experiments in the current study were designed to avoid such confounders; kynurenine was dissolved in PSS only and addition of kynurenine 10 mmol/L stock solution (pH 7.6) to CO_2_ perfused organ baths containing buffered saline solutions did not change pH.

We also provide the first evidence that the vasorelaxant effects of kynurenine are preserved in human arteries obtained from subjects with hypertension, in this case, preeclampsia. Among women with preeclampsia recruited to this study, over half required delivery before 34 weeks’ gestation, and 60% of babies weighed less than the customized 10th percentile, indicating a skew towards a severe disease phenotype. Despite in vivo evidence of vascular dysfunction, demonstrated by hypertension requiring therapy, arteries isolated from women with preeclampsia relaxed substantially in response to kynurenine and were less responsive to U46619 following kynurenine treatment. Although relaxation of preconstricted arteries in response to midrange kynurenine doses was reduced in arteries from women with preeclampsia compared with normotensive women, direct comparison between these unmatched cohorts requires caution. Differences in kynurenine-induced relaxation could be related to observed baseline differences in U46619-induced constriction. Furthermore, numerous sources of clinical variation existed between groups, including disease status, gestational age, and drug exposures (eg, antihypertensives, magnesium sulfate, corticosteroids). Such variables could confound the vascular response to kynurenine. Due to the large number of clinical variables, the current sample size is inadequate to identify clinical predictors of the vascular response to kynurenine.

The substantial and reproducible vasorelaxant effects of kynurenine in multiple vascular beds are encouraging for the possibility of a systemic effect of kynurenine on BP. In rats, similar in vitro responses to kynurenine were observed in resistance arteries^1^ and subsequently, systemic BP effects have been demonstrated in response to intravenous kynurenine treatment^1^ or oral tryptophan supplementation.^10^ The potential for kynurenine pathway manipulation to be applied clinically as a future adjunct or alternative antihypertensive strategy for pregnant and nonpregnant patients merits further investigation. Research in this area remains in its infancy. Although studying isolated effects on arteries and VSMCs offers advantages for identifying the role of specific cellular mechanisms, this study is limited by its reliance on in vitro studies. Translation of these findings will require extensive investigation due to the current paucity of in vivo evidence of kynurenine pathway manipulation to help address issues such as dosing and off-target effects.

Determining the mechanism of kynurenine-induced vasorelaxation was a vital first step for therapeutic translation. To elucidate this mechanism, we used a systematic approach combining myography, high-speed confocal microscopy of pressurized arteries, and patch clamp electrophysiology of freshly isolated VSMCs, all using omental arteries from healthy participants. First, kynurenine-mediated vasorelaxation was confirmed to be endothelium-independent.^1,9^ Further myography protocols indicated that kynurenine-induced relaxation via activation of VSMC BK_Ca_ channels, with abolition of kynurenine-induced relaxation by 2, highly specific, BK_Ca_ inhibitors with distinct binding locations,^20,21^ providing confidence in the specificity of this mechanism. Myography and electrophysiology experiments suggest that kynurenine does not act via other K^+^ channels, in fact showing a small reduction in the paxilline-resistant outward current following kynurenine treatment. These data contradict a previous report implicating type-7 K_v_ channels as the mediators of kynurenine-induced relaxation in human omental arteries.^9^ In our myography experiments, which included control-treated arteries, reduced relaxation in linopirdine-treated arteries occurred in response to low doses of kynurenine or vehicle, indicating nonspecific inhibition of spontaneous arterial relaxation rather than inhibition of kynurenine-induced effects.

The GC and AC pathways, which can both regulate BK_Ca_ activity, have previously been implicated in kynurenine-mediated vasorelaxation.^1^ However, in this study, inhibition of the GC/cGMP/PKG pathway by 3 mechanistically distinct inhibitors had no functional effect on relaxation in response to kynurenine. Although AC inhibition consistently caused a modest reduction in kynurenine-induced relaxation, comparable to that reported by Wang et al,^1^ the large remaining relaxation indicates additional mechanisms. We, therefore, examined the effects of kynurenine on Ca^2+^ sparks, which are the principal driver of BK_Ca_ channel–mediated vasodilation under steady-state conditions. In pressurized arteries, studied using high-speed confocal microscopy, kynurenine significantly increased Ca^2+^ spark frequency. However, patch clamp electrophysiology was not consistent with this. Using the perforated patch approach, it is possible to maintain an intact cytoplasm and thus preserve the inherent Ca^2+^ spark activation of plasmalemmal BK_Ca_ channels which is seen as STOCs. If kynurenine were to mediate its vasodilatory effects predominantly via an increase in Ca^2+^ spark frequency, one would anticipate an increase in STOC frequency. However, this was not the case; kynurenine merely led to an increase in STOC amplitude. We, therefore, studied the BK_Ca_ channel directly, using a whole-cell approach with dialysis of the cytoplasm and, therefore, removal of any effects on the BK_Ca_ channel by Ca^2+^ sparks. Using this method, we observed that kynurenine increased the outward current through the BK_Ca_ channel directly. Taken in conjunction with the observation that kynurenine vasodilation is inhibited by paxilline and iberiotoxin but not by ryanodine, we propose that the predominant vasodilatory mechanism by which kynurenine initiates its effects is a direct effect on the BK_Ca_ channel. An additional but lesser contributory effect via an increase in Ca^2+^ spark frequency is likely to occur in parallel and may reflect a diverse and pleiotropic influence of kynurenine and its metabolites on the vasoregulatory Ca^2+^ spark–BK_Ca_ channel axis in VSMC. Further evaluation of this is necessary to clarify precise mechanisms. A summary of the vascular effects of kynurenine is shown in Figure IV in the Data Supplement.

Data may indicate kynurenine activates BK_Ca_ via effects on multiple targets, but we cannot exclude the possibility of effects by multiple vasoactive factors due to either contamination, modification, or metabolism within commercially-available kynurenine. This is a source of on-going speculation, as the group that first demonstrated the vasorelaxant effects of commercially-available Kyn^1^ subsequently failed to recreate their findings using freshly purified kynurenine.^6^ Similarly, in vitro, purified kynurenine has low potency for activating the transcription factor AhR (Aryl hydrocarbon receptor),^22^ through which it mediates its immune effects. However, aging of kynurenine preparations spontaneously increases AhR activation alongside accumulation of newly identified trace kynurenine derivatives termed trace-extended aromatic condensation products.^22^ Understanding of the kynurenine pathway is rapidly evolving due to improved analytical tools and new interest in potential human therapeutics. Recently, a novel kynurenine pathway intermediary was identified which directly dimerizes PKG to cause vasorelaxation: cis-hydroperoxide ([2S,3aR,8aR]-3a-hydroperoxy-1,2,3,3a,8,8a-hexahydropyrrolo[2,3-b]indole-2-carboxylic acid). Cis-hydroperoxide ([2S,3aR,8aR]-3a-hydroperoxy-1,2,3,3a,8,8a-hexahydropyrrolo[2,3-b]indole-2-carboxylic acid), produced by indoleamine 2,3-deoxygenase from tryptophan and singlet oxygen in conditions of oxidative stress, spontaneously degrades to the kynurenine precursor N-formylkynurenine.^6^ As a highly unstable, upstream metabolite of kynurenine, cis-hydroperoxide ([2S,3aR,8aR]-3a-hydroperoxy-1,2,3,3a,8,8a-hexahydropyrrolo[2,3-b]indole-2-carboxylic acid) is very unlikely to be present as a contaminant in commercially available kynurenine. Our finding that kynurenine-induced relaxation is unaffected by KT5823, an ATP-competitive inhibitor of PKG, provides further confidence that vasorelaxant effects reported here are unrelated to cis-hydroperoxide ([2S,3aR,8aR]-3a-hydroperoxy-1,2,3,3a,8,8a-hexahydropyrrolo[2,3-b]indole-2-carboxylic acid). Most downstream kynurenine pathway metabolites have previously been demonstrated to have no vasorelaxant effects (kynurenic acid,^1,8^ 3-hydroxykynurenine,^1^ 3-hydroxyanthranilic acid,^1^ or xanthurenic acid^8^), although experiments with quinolinic acid have been conflicting.^1,8^ Kynurenine (>97% high-performance liquid chromatography [HPLC]) used in this study and elsewhere^1,8,9^ was obtained from Sigma but no further information on the source or manufacturing could be obtained. This study cannot retrospectively investigate whether vasorelaxation in human arteries used over a 7-year period was attributable to either a spontaneous modification of molecular kynurenine or a contaminant. Future work, which must be performed in collaboration with biochemists, will attempt to address these questions. Identification of individual vasoactive factors that can independently activate BK_Ca_ channels or ryanodine receptors would be useful for more targeted therapeutics. In particular, a factor capable of causing these vasorelaxant effects at trace concentrations may be advantageous for future translation.

Kynurenine concentrations used experimentally greatly exceed kynurenine plasma levels, which are around 1 µmol/L during and outwith pregnancy,^12,13,23^ increasing in pathologies including obesity (2 µmol/L),^12^ trauma (2–3 µmol/L),^4^ pulmonary hypertension (3–4 µmol/L),^7^ and sepsis (20 µmol/L),^11^ although surprisingly not in preeclampsia.^13,23^ However, the ability of endogenous levels of kynurenine pathway metabolites to cause systemic BP changes is established in animal models of inflammation^1,5^ and inferred in human studies where changes in endogenous kynurenine pathway activity inversely correlate with systemic BP.^4,11,12^ Serum kynurenine concentrations exceeding these physiological levels can be achieved in pregnant mice by oral dosing with kynurenine.^24^ Although there is no evidence relating to kynurenine dosing in humans, oral tryptophan (5 g/day for 21 days) achieves a sustained 3- to 4-fold increase in urinary excretion of kynurenine, without affecting dietary intake, anthropometry, or mood.^25,26^ Tryptophan dosing is a less appealing therapeutic strategy than kynurenine due to likely effects on serotonin pathways,^27^ although potential upstream effects of kynurenine treatment on serotonin/melatonin production will also require consideration.

The diverse, and often competing, physiological roles of kynurenine pathway metabolites make extrapolation of in vivo effects of kynurenine pathway manipulation difficult. As it is activated by inflammation, kynurenine pathway activity is increased in several cardiovascular pathologies, but there is no consensus whether net effects of kynurenine pathway activity are beneficial or detrimental (reviewed by Song et al^28^). In several pathologies, it has been proposed that kynurenine pathway activation acts as a negative feedback mechanism to constrain the inflammatory response or in the case of pulmonary hypertension to promote pulmonary artery relaxation in response to increased pressure.^7^ It could be anticipated that kynurenine use in vivo could confer additional vascular benefits via several mechanisms: (1) downstream conversion to potent antioxidants such as 3-hydroxykynurenine and 3-hydroxyanthranilic acid^29^; (2) generation of NAD^+^ leading to activation of Sirtuin receptors^30^ and; (3) prevention of NAD^+^ depletion following poly-ADP ribose polymerase activity.^2^ Uncertainties about the systemic effects of kynurenine on the vasculature can be extended to other systems, most notably neurological^31,32^ and immune. Observational and experimental evidence suggests kynurenine pathway manipulation may have immune effects by activating the AhR transcription factor and promoting T-cell tolerogenicity^22,33^; although in theory, this may be beneficial in the case of preeclampsia,^19^ this is currently speculative and requires further investigation.

This work highlights a gap in knowledge surrounding the role of VSMC BK_Ca_ channels in pregnancy. To our knowledge, this is the first time Ca^2+^ sparks and STOCs have been recorded from omental arteries from pregnant women. In response to kynurenine, there was a significant increase in the frequency of Ca^2+^ sparks but not STOCs. This may indicate a predominant effect of kynurenine on BK_Ca_ channels rather than Ca^2+^ spark frequency. An alternative possibility is that pregnancy may induce weak coupling of Ca^2+^ sparks and BK_Ca_ activation. Although uterine arteries are known to have altered BK_Ca_ expression, activity, and regulation in pregnancy,^34,35^ the role of maternal BK_Ca_ has not been assessed outside the uterine circulation in normal pregnancy, or in the context of pregnancy pathologies, such as preeclampsia. Preservation of the maximal relaxatory response to kynurenine and NS11021 in the hypertensive cohort indicates VSMC BK_Ca_ channels themselves may offer an effective therapeutic target for preeclampsia, despite the vascular dysfunction present in this disease.

In the current study, corrections were made for multiple comparisons within each data set, but corrections were not made for multiple hypothesis testing across all experiments, which is a limitation. Some mitigation is provided by large sample sizes and the use of multiple inhibitors/experimental approaches to corroborate conclusions. The potential contribution of each woman to multiple data sets may also impact findings, but this pragmatic approach was necessary for the efficient use of biopsies, which permitted all experiments to be completed using human arteries. Electrophysiology and Ca^2+^ spark data have been analyzed according to standard approaches. However, it should be acknowledged that this approach could not account for the nonindependence of data from different arteries/cells contributed by the same woman.

The treatment of hypertension in pregnancy presents unique considerations due to the presence of 2 patients with different requirements, and 2 separate circulations, which contribute to the maintenance of adequate placental exchange. It is essential that vasorelaxant effects upon systemic maternal arteries also occur in the uterine vascular bed, as has been shown for kynurenine, to maintain uterine perfusion as systemic vascular resistance falls. Meanwhile, vasorelaxation in the fetoplacental circulation, which could lead to fetal hypotension, may be detrimental to fetal wellbeing and necessitate expedited delivery. This is particularly important for agents such as kynurenine, which are known to readily cross the placenta and become selectively enriched in the fetal circulation.^24^ Data presented here show that vasorelaxant effects of kynurenine are relatively selective for maternal arteries, with no direct relaxatory effect on placental arteries, and attenuation of U46619 dose-contraction curves only at higher doses; this is further encouraging for its application in pregnancy. However, these data must be interpreted with caution because the chorionic plate arteries tested cannot be assumed to be representative of smaller placental stem arteries/arterioles. The use of any drug in pregnancy necessarily has substantial safety considerations and extensive evidence of short-term and long-term safety, in addition to antihypertensive efficacy, will be required from in vitro and animal studies before the use of kynurenine in human pregnancy. In particular, assessment of potential effects on fetal neurodevelopment will be critical due to the linked regulation of kynurenine and serotonin pathways^32^ and the proposed involvement of kynurenine pathway dysregulation in schizophrenia.^31^ The absence of placental artery relaxation in response to kynurenine or NS11021 at doses tested here, suggests BK_Ca_ have a limited functional role in determining placental chorionic plate vascular tone. This is interesting considering the near-ubiquitous functional role of BK_Ca_ channels in excitable tissue. However, it is consistent with previous work from our group which confirms the presence of BK_Ca_ in VSMCs of placental arteries, although with a limited functional effect of BK_Ca_ inhibitors compared with other K^+^ channels.^36^ Although one study has reported chorionic plate artery relaxation in response to the BK_Ca_ activator NS1619, this agent is less specific than NS11021 and can also activate K_v_ channels,^37^ which are important regulators of chorionic plate artery tone.^36^

We conclude that kynurenine acts to boost the Ca^2+^ spark–BK_Ca_ channel vasoregulatory axis within VSMCs of resistance arteries obtained from normotensive women and this effect is maintained in arteries from women with preeclampsia. The data warrants further investigation of the potential to exploit this endogenous vasorelaxant as a novel antihypertensive therapy.

## Sources of Funding

This work was supported by a Wellbeing of Women Entry-level Scholarship (S.A. Worton; ELS083), a Medical Research Council / Royal College of Obstetricians and Gynaecologists Clinical Research Training Fellowship (S.A. Worton; MR/N020685/1), and a British Heart Foundation project grant (A. Greenstein; PG/18/7/33535).

## Disclosures

None.

## Supplemental Materials

Expanded Materials and Methods

Data Supplement Figures I–IV

References 38–40

Major Resources Table

## Supplementary Material



## Novelty and Significance

### What Is Known?

- In animal models of infection and inflammation, conversion of tryptophan to kynurenine causes vasorelaxation which contributes to the development of hypotension.
- In the hypertensive disorder of pregnancy, preeclampsia, impaired relaxation of maternal small arteries is a key contributor to hypertension and end-organ dysfunction.
