# Supplementary figures and images for: Kynurenine Relaxes Arteries of Normotensive Women and Those With Preeclampsia
Source: Circ Res. 2021 Mar 3;128(11):1679–93. doi: 10.1161/CIRCRESAHA.120.317612 (PMC8154175; doi:10.1161/CIRCRESAHA.120.317612)

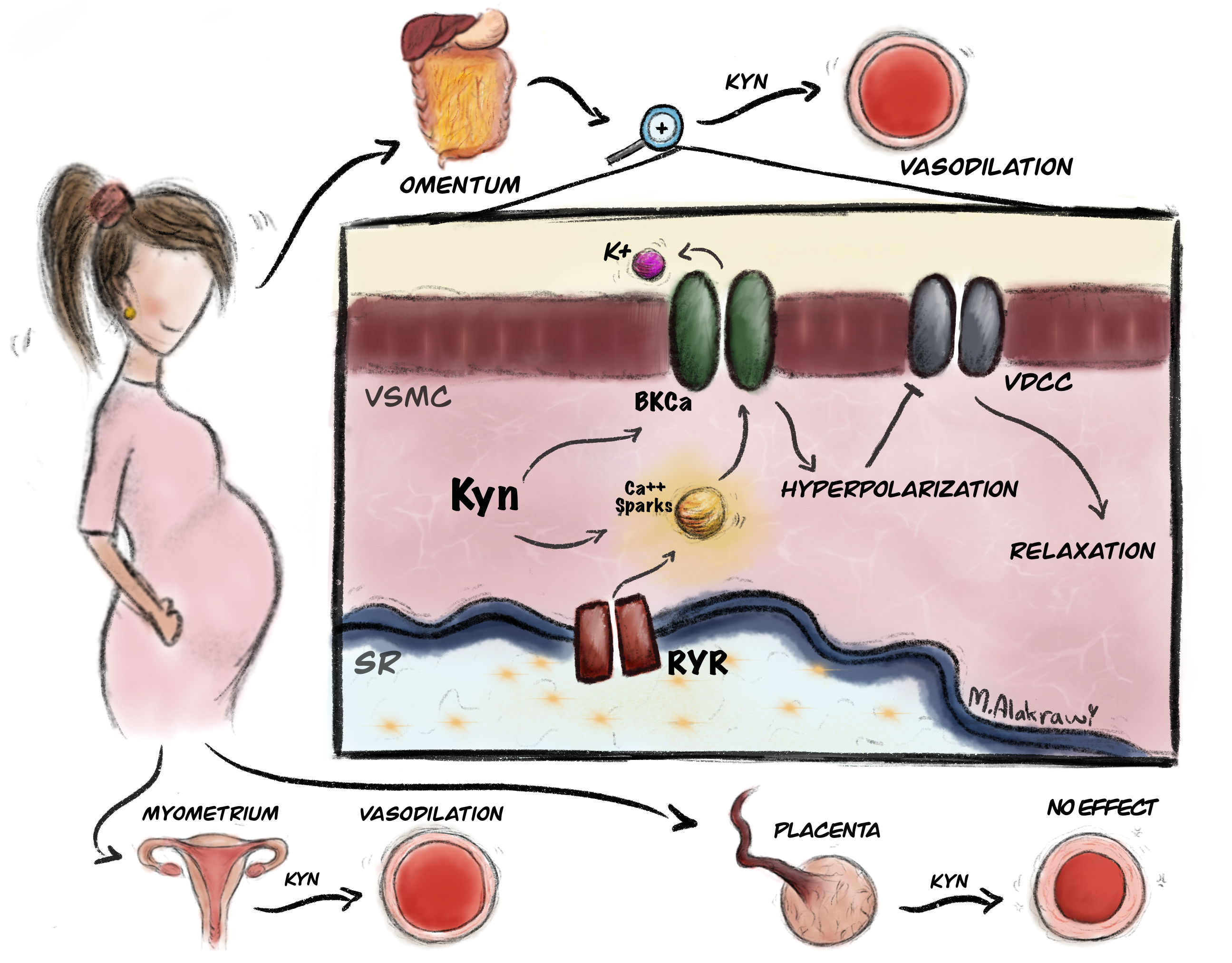

Supplement: Supplementary file 1 [file res-128-1679-s001.tif]
