# Supplementary material for: Kynurenine Relaxes Arteries of Normotensive Women and Those With Preeclampsia
Source: Circ Res. 2021 Mar 3;128(11):1679–93. doi: 10.1161/CIRCRESAHA.120.317612 (PMC8154175; doi:10.1161/CIRCRESAHA.120.317612)
Supplement: Supplementary file 2 [file res-128-1679-s002.pdf]

SUPPLEMENTAL MATERIAL

Expanded materials and methods

The data that support the findings of this study are available from the corresponding author upon reasonable request.

Participants

Omental, myometrial or placental samples were obtained with prospective, written consent in accordance with ethics committee approval (National Research Committee North West – Haydock Board (08/H1010/55(+5))). For some women, samples were obtained from more than one vascular bed. Hypertensive women had a clinical diagnosis of preeclampsia, defined according to International Society for the Study of Hypertension in Pregnancy guidance[38] as the new onset of hypertension (systolic BP ≥140 mmHg or diastolic ≥90 mmHg on more than one occasion) after 20 weeks of pregnancy in the presence of either i) proteinuria, ii) maternal organ dysfunction, or iii) uteroplacental insufficiency. Women were excluded if they had a significant medical co-morbidity, including: pre-existing hypertension, diabetes, renal disease, cardiac disease, autoimmune conditions, haematological disorder, or for fetal abnormality. Additional exclusion criteria for normotensive women included body mass index >35 kg/m^2, any gestational hypertensive disorder, delivery indicated for suspected/confirmed fetal growth restriction, or infant individualised birthweight ratio <3rd centile (Customized Weight Centile Calculator version 5.12/6.2 2009 downloaded from www.gestation.net).

Tissue samples

Biopsies were obtained from the maternal omentum (~2x2x0.5 cm) or myometrium (~1 cm^3; from the upper edge of the uterine incision) during Cesarean section, or were sampled from the placental chorionic plate within 30 minutes of Cesarean or vaginal delivery. Resistance arteries (100-500 µm) were dissected from omental, myometrial and placental biopsies in cold physiological saline solution (PSS; (in mmol/L): 119 NaCl, 25 $NaHCO_3$, 4.69 KCl, 2.4 $MgSO_4$, 1.6 $CaCl_2$, 1.18 KH_2PO_4, 6.05 glucose, 0.034 Ethylenediaminetetraacetic acid; pH 7.4). Isolated omental arteries from each woman were used for wire myography, Ca^{2+} spark imaging, electrophysiology studies or a combination of these depending on experimental requirements at the time of recruitment.

Wire Myography

Resistance arteries were mounted onto parallel wires in a Danish Myotechnology M610 wire myograph (Danish Myotech, Aarhus, Denmark), and individually normalised to an internal pressure of 0.9 L13.3 kPa (omental and myometrial)[39] or L5.1 kPa (placental)[40], as previously described. In a subset of omental experiments (N=7), the vascular endothelium was mechanically removed with a human hair. Throughout all experiments, arteries were maintained at 37 °C in PSS perfused with CO_2 (5 %) and O_2 (omental and myometrial 21 %; placenta 5 %, normal placental oxygenation).[39,40] Arteries were allowed to equilibrate for 20-30 minutes prior to experimentation. Smooth muscle response to 120 mmol/L K^+ solution (KPSS; (in mmol/L): 11 NaCl, 25 $NaHCO_3$, 120 KCl, 2.4 $MgSO_4$, 1.6 $CaCl_2$, 1.18 KH_2PO_4, 6.05 glucose, and 0.034 Ethylenediaminetetraacetic acid; pH 7.4) was measured twice at the beginning and end of experiments. For all arteries, baseline concentration-response curves were constructed for the synthetic thromboxane-A_2 receptor agonist U46619 (10^{-10}-$10^{-5.7}$ mol/L; Merck Chemicals, Nottingham, U.K.); arteries were

allowed 2-5 minutes to reach a stable plateau prior to addition of the next dose and maximal constriction recorded for each dose. A single (maximal) dose of the endothelium-dependent vasorelaxant bradykinin (10 μmol/L) was added and the maximal relaxation measured over 5 minutes. All arteries were washed to baseline and allowed to equilibrate prior to further experimentation. Arteries were blindly allocated to treatment conditions by an independent researcher.

Relaxation of pre-constricted arteries: Vessels were treated with individually calculated EC_{80} concentrations of U46619 and, as required, supplemented to achieve a stable sub-maximal constriction. Kyn (10 mmol/L in PSS) or PSS (vehicle control) was added at 2 minute intervals to construct concentration-response curves (0.05-3 mmol/L; dose range as per Wang *et al*[1]). To reduce dilutional effects, Kyn or vehicle control solutions for each vessel were warmed to 37 ˚C and individually spiked with doses of vasoconstrictors/inhibitors corresponding to the organ bath. After the final dose, relaxation in response to bradykinin 10 μmol/L was assessed over 5 minutes. For a subset of women, control-treated arteries were washed out and subsequently preconstricted (U46619 EC_{80}) to assess relaxation in response to the BK_{Ca} channel activator NS11021 (10 μmol/L) or vehicle control (PSS/ethanol; final ethanol concentration 0.1%). To investigate the mechanism of Kyn-induced effects, arteries were treated with inhibitors prior to submaximal constriction, as follows; (i) tetraethylammonium chloride (TEA) 5 mmol/L, 30 minutes; (ii) linopirdine 10 μmol/L, 30 minutes; (iii) 4-aminopyridine 1 mmol/L, 30 minutes; (iv) paxilline 1 μmol/L, 15 minutes; (v) iberiotoxin (Latoxan, Portes lès Valence, France) 100 nmol/L, 15 minutes; (vi) ryanodine (Tocris bioscience, Abingdon, U.K.) 20 μmol/L, 30 minutes; (vii) 1H-[1,2,4]oxadiazolo[4,3,-a]quinoxaline-1–one (ODQ; Cayman Chemical, Ann Arbor, Michigan) 10 μmol/L, 30 minutes; (viii) Rp-8-CPT-cGMPS 1

mmol/L, 30 minutes; (ix) KT5823 2 µmol/L, 30 minutes; (x) 9-(Tetrahydro-2-furanyl)-9H-purin-6-amine (SQ22,536) 100 µmol/L, 30 minutes. Inhibitor experiments were undertaken in an order dictated by the evolving scientific rationale, but with overlap between inhibitors. Where a sufficient number of arteries was available, arteries from a single woman were used to test the effects of more than one inhibitor, such that vessels from each participant may have contributed data to multiple data sets. For inhibitors that did not have any effect on Kyn-induced relaxation, validation experiments were undertaken in omental arteries to confirm their effectiveness at the stated concentration/duration against target pathways, as follows: (1) Arteries incubated with linopirdine, ODQ, Rp-8-CPT-cGMPS and KT5823 were preconstricted (U46619 EC_{80}) and relaxation assessed in response to incremental doses of well-established vasorelaxants acting via the target pathway (or vehicle control). Linopirdine-treated vessels were exposed to the K_v7 activator retigabine (10^{-8}–10^{-5} mol/L), whilst arteries treated with guanylate cyclase pathway inhibitors (ODQ, Rp-8-CPT-cGMPS and KT5823) were exposed to sodium nitroprusside (10^{-9}-10^{-6} mol/L). (2) To demonstrate an effect upon K_v channels, arteries were partially depolarised by bathing in PSS containing K^+ 15 mmol/L. After 5 minutes to equilibrate, 4-aminopyridine or vehicle were added to the bath and the constriction in response to K_v channel inhibition recorded as the maximal constriction over the subsequent 5 minutes. (3) Arteries treated with ryanodine or vehicle control had bathing solutions replaced with PSS containing 5 mmol/L caffeine and the maximal amplitude of the evoked transient constriction was measured.

Effect of Kyn on U46619-induced constriction: Arteries were incubated in PSS at 37 ˚C in 5% CO_2 in the presence of Kyn (1 or 6 mmol/L) or PSS only (vehicle

control). After 1 hour, concentration-response curves to U46619 (10^{-10}-$10^{-5.7}$ mol/L) were repeated in the continued presence of Kyn or PSS.

Analysis and Statistics: Arterial tension was measured within Myodata 2.02 software (National Instruments Corporation, Austin, Texas) and statistics performed using GraphPad Prism 7 (GraphPad Software, San Diego, California). For each vascular bed, arterial traces which were representative of the mean performance of analysed arteries were selected for inclusion in figures. Treated and control artery groups were compared by paired t-tests or Wilcoxon signed-rank tests, depending on results of the Shapiro-Wilk normality test. All p-values are shown to 2 s.f. and p-values <0.01 are written in scientific notation. Where precise p-values were below the reporting threshold of GraphPad Prism ($p<1x10^{-15}$ for Sidak's test and $1x10^{-4}$ for Dunnett's test) precise values were calculated from effect size and 95% Confidence Intervals to satisfy the journal's statistical reporting standards.

Relaxation: Relaxation in response to bradykinin 10 µmol/L or NS11021 10 µmol/L was recorded 5 minutes after addition of a maximal dose. For concentration-relaxation curves, 'constriction' at each dose was calculated as mean remaining constriction during 2 minute dose intervals, and expressed as a proportion of maximal response to EC_{80} dosing. Arteries were excluded at analysis if U46619 EC_{80} pre-constriction was ≤40% of maximal constriction or relaxation >50% occurred during the first dose interval. Experiments were only included if data was available for at least one control and one vehicle treated artery. Duplicate/triplicate arteries were averaged for each woman; samples sizes are expressed as n=number of vessels, N=number of participants and where only N is given, N=n. Concentration-response curves for relaxation data are shown as mean ± SEM and analysed by 2-way repeated measures (RM) analysis of variance (ANOVA) with Sidak's (2 groups)

or Dunnett's (>2 groups) multiple comparison tests, in a paired comparison between arteries from the same woman. Relaxation data were compared by 2-way ANOVA regardless of the outcome of the Shapiro-Wilk normality test due to the absence of an equivalent non-parametric test that can recognise the interaction between variables; for transparency, where a 2-way ANOVA test is used, it is stated in the figure legend if data are not normally distributed. For multiple comparison tests, all p-values are adjusted for the number of pairwise comparisons made and p-values <0.05 are stated on figures. **U46619-induced constriction:** Post-treatment constriction to U46619 was expressed as a proportion of pretreatment maximal response to U46619 $10^{-5.7}$ mol/L, to adjust for baseline contractile function. Data are shown as mean ± SEM with fitted curves derived by four-parameter non-linear regression. Post-treatment constriction was compared between groups using 2-way RM ANOVA with Dunnett's multiple comparison tests between treated and control-treated arteries; multiple comparisons were not made between groups treated with different doses of Kyn. Normally-distributed and non-normally-distributed data were analysed the same, as previously described. For each artery $-logEC_{50}$ (pEC_{50}) was interpolated by non-linear regression; arteries for which EC_{50} was outside 10^{-10}-$10^{-5.7}$ mol/L were excluded from this analysis (17/309 arteries). The constriction of each artery was adjusted for its baseline response by calculating the change in pEC_{50} (ΔpEC_{50}). Due to sample sizes, analysis did not assume a normal distribution and ΔpEC_{50} are described as median (IQR); treated arteries were compared to paired controls from the same woman using Wilcoxon signed-rank tests.

Ca^{2+} spark imaging

To load with the Ca^{2+} indicator dye, resistance arteries were incubated in the dark for 45 minutes at room temperature in a HEPES-based solution containing (in mmol/L) 134 NaCl, 6 KCl, 1 $MgCl_2$, 2 $CaCl_2$, 7 glucose, and 10 HEPES (pH 7.4; NaOH) supplemented with Fluo-4 AM (10 µmol/L; Thermofisher Scientific, Waltham, Massachusetts) and pluronic acid (0.05% v/v). Arteries were mounted on glass micropipettes in an arteriography chamber, pressurized to 80 mmHg, and superfused with PSS. Imaging PSS was aerated with a 5 % CO_2, 21 % O_2, balance N_2 gas mixture and warmed to 37 °C. Following 15 minutes equilibration, Fluo-4–loaded arteries were excited by illuminating at 488 nm using a solid-state laser, and fluorescence emission was collected above 510 nm. Images (131 × 131 µm) were recorded every 18.9 ms (53 frames/second) using a 60× water immersion objective (final magnification, 600×; NA1.2) attached to a Nikon Eclipse TE - 2000U microscope. Imaging was repeated following addition of Kyn (1 mmol/L) to perfusate.

Analysis and statistics: Ca^{2+} sparks in the vascular smooth muscle layer were automatically detected using SparkAn, software which identifies temporally delineated increases in fractional fluorescence (F) of greater than 26% above baseline fluorescence levels (F_0) in defined regions of interest (ROIs) of 1.5 × 1.5 µm (7 × 7 pixels). Subcellular Ca^{2+} changes within ROIs are expressed as F/F_0. An average of frequency, amplitude, duration, rise and decay time was taken from 1-3 recordings per vessel. Where readings were obtained from multiple vessels from a single woman these data were treated as independent for the purpose of statistical analysis. Data were not normally distributed (Shapiro-Wilk normality test) and were analysed by a non-parametric test; measurements pre- and post-Kyn for each vessel were compared in a paired analysis by Wilcoxon-signed rank tests.

Single cell isolation

Vascular smooth muscle cells were isolated from omental resistance arteries in a dispersal physiological saline solution (DPSS; (in mmol/L): 55 NaCl, 80 Na-glutamate, 6 KCl, 2 $MgCl_2$, 10 glucose and 10 HEPES; pH 7.3) supplemented with papain (2.5 mg/ml; Worthington Biochemicals, Lakewood, New Jersey), dithioerythritol (1 mg/ml) and bovine serum albumin (10 mg/ml) at 37 °C for 17 minutes, washed three times with DPSS, and further incubated for 6 minutes at 37 °C in DPSS containing collagenase F (0.6 mg/ml) and 0.1 mmol/L $CaCl_2$. Digested arteries were triturated to liberate VSMCs, which were stored in ice-cold DPSS and studied within 6 hours.

Patch-clamp electrophysiology

All recordings were performed at room temperature (22 °C). VSMCs were transferred to a recording chamber and allowed to adhere to glass coverslips for 10-20 minutes. Recording electrodes (3 to 5 MΩ) were pulled and polished. Currents recorded from VSMCs were performed in perforated-patch and ruptured whole cell patch configuration. All currents were recorded using an AxoPatch 200B amplifier equipped with an Axon CV 203BU headstage (Molecular Devices, San Jose, California). Currents were filtered at 1 kHz, digitized at 40 kHz, and stored for subsequent analysis. Clampex and Clampfit (version 10.2; Molecular Devices) were used for data acquisition and analysis, respectively.

Spontaneous transient outward currents (STOCs): For perforated-patch whole-cell recordings, amphotericin B (40 µM) was included in the pipette solution to allow electrical access. Perforation was deemed acceptable if series resistance was less than 40 MΩ. Spontaneous transient outward currents (STOCs) were recorded before

and after addition of Kyn 1 mmol/L in a bathing solution containing (in mmol/L): 134 NaCl, 6 KCl, 1 $MgCl_2$, 2 $CaCl_2$, 10 glucose, and 10 HEPES (pH 7.4). The pipette solution contained (in mmol/L): 110 K-aspartate, 1 $MgCl_2$, 30 KCl, 10 NaCl, 0.005 EGTA, and 10 HEPES (pH 7.2). STOCs were recorded from VSMCs that were voltage-clamped at – 20 mV. **Analysis and statistics:** Due to small sample sizes, data were not assumed to be normally distributed and were analysed using non-parametric tests. For each individual VSMC, the frequency (Hz) and maximum amplitude (pA) of STOCs obtained were compared before and after Kyn treatment by Wilcoxon signed-rank tests. Where readings were obtained from multiple cells from a single woman these data were treated as independent for the purpose of statistical analysis.

Whole cell: Ruptured whole-cell K^+ currents were recorded using a step protocol (−100 to +100 mV in 20-mV steps for 500 ms) from a holding potential of −30 mV. Sequential recordings were made in each artery (i) at baseline, (ii) following addition of Kyn 1 mmol/L or vehicle control (bathing solution), and (iii) following addition of paxilline (1 µmol/L). The VSMC bathing solution contained (in mmol/L): 134 NaCl, 6 KCl, 10 glucose, 2 $CaCl_2$, 1 $MgCl_2$ and 10 HEPES (pH 7.4). The pipette solution contained (in mmol/L): 140 KCl, 1.9 $MgCl_2$, 0.075 Ca^{2+}, 0.1 EGTA, and 2 Na_2ATP, and 10 HEPES (pH 7.2; KOH). **Analysis and statistics:** Whole-cell BK_{Ca} currents (corrected for cell capacitance) were isolated from other K^+ currents as a subtraction current before and after the addition of paxilline. Paxilline-sensitive current-voltage characteristic curves were generated using mean values obtained from the last 50 ms of each voltage step. Where readings were obtained from multiple cells from a single woman these data were treated as independent for the purpose of statistical

analysis. Curves were compared by 2-way RM (one-factor) ANOVA with Sidak's multiple comparison tests.

Materials: Please see the Major Resources Table in the Supplemental Materials. Unless otherwise stated, reagents were obtained from Sigma-Aldrich Company Ltd (Poole, UK). Myography reagents were diluted in PSS where possible and where additional solvents were required, the final concentration in organ baths was ≤0.2%.

Supplemental Figures and Figure Legends

Supplemental Figure I

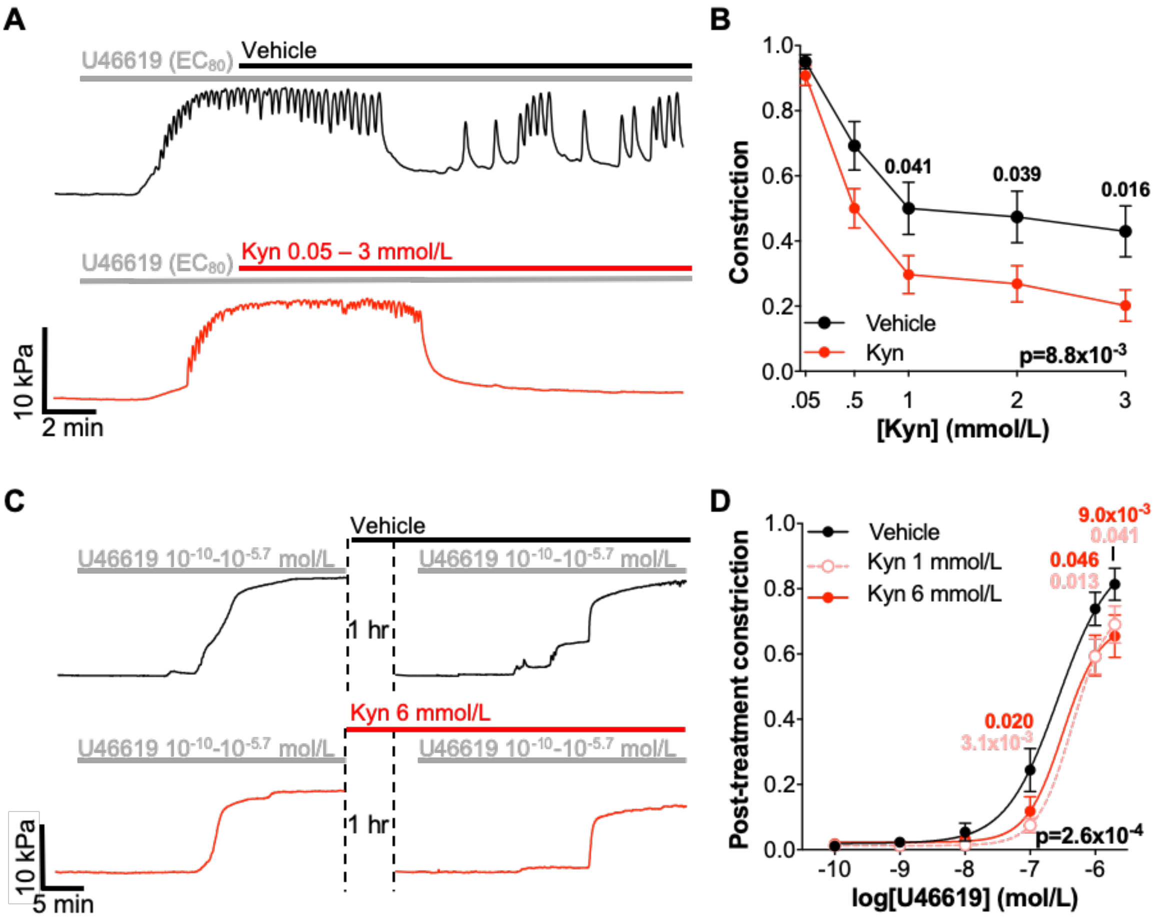

Supplemental Figure I: Kyn causes vasorelaxation and reduces vasoconstriction in myometrial arteries. (**A**) Representative myography traces of preconstricted myometrial arteries from the same woman treated with increasing doses of vehicle (PSS; black) or Kyn (0.05-3 mmol/L; red) **(B)** Cumulative post-treatment concentration-relaxation curves for preconstricted myometrial arteries (n=26-29, N=20). Kyn- and vehicle-treated arteries compared by 2-way RM ANOVA with Sidak's multiple comparison tests. Data are not normally distributed. **(C)** Representative myography traces of individual myometrial arteries constricted with increasing doses of U46619 (10^{-10}–$10^{-5.7}$ mol/L) before and after 1 hour treatment with vehicle (PSS; black) or Kyn 6 mmol/L (red). **(D)** Cumulative concentration-response curves for U46619 (10^{-10}–$10^{-5.7}$ mol/L) following 1 hour treatment with Kyn 1 mmol/L or 6 mmol/L or vehicle control (N=21-25). Constriction relative to pre-treatment maximum. Data are not normally distributed. Curves compared by 2-way RM ANOVA with Dunnett's multiple comparison to control-treated arteries.

Supplemental Figure II

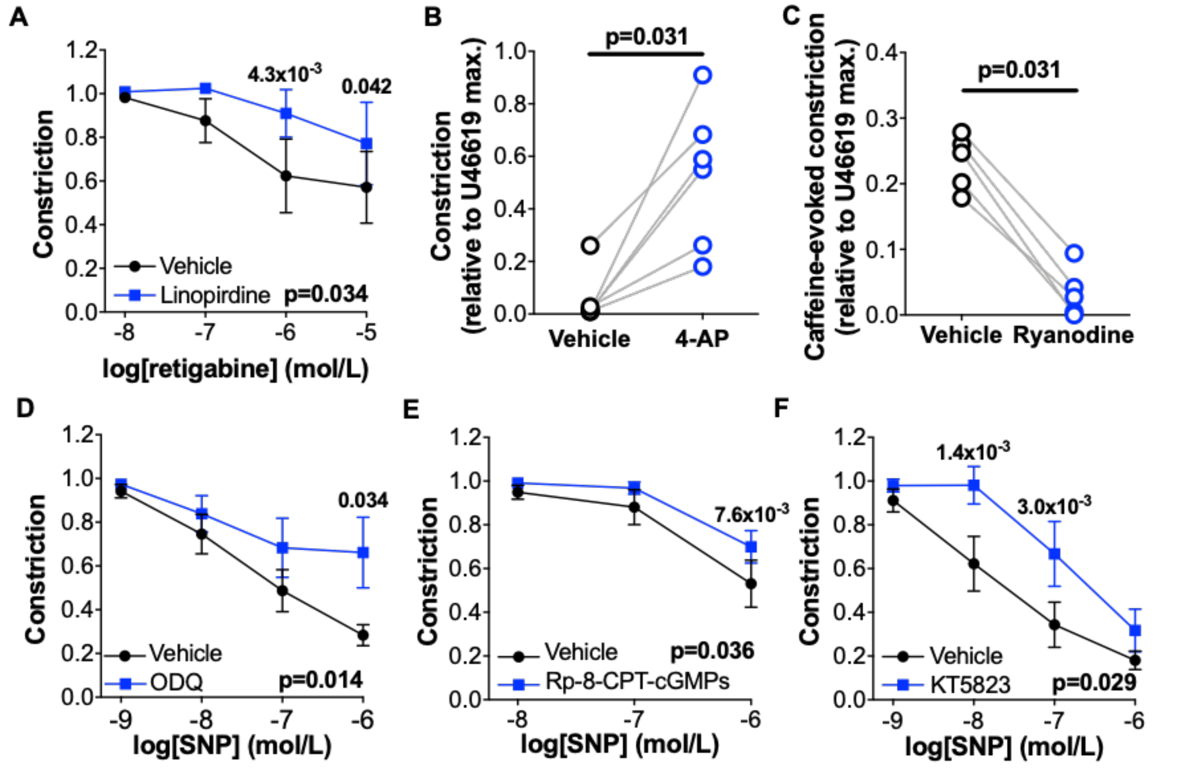

Supplemental Figure II: Confirmation of the effects of inhibitory agents on target pathways in omental arteries from pregnant women. (A) Relaxation of preconstricted arteries in response to Kv7 activator retigabine (10^{-8}–10^{-5} mol/L) following pre-treatment with vehicle (PSS; 0.05% DMSO) or linopirdine 10 μmol/L (n=9, N=5). **(B)** Constriction of partially-depolarised arteries (bathed in 15 mmol/L K^+ solution) in response to vehicle (HEPES-based solution) or 4-aminopyridine 1 mmol/L (4-AP; N=6). Wilcoxon signed-rank test. **(C)** Maximal constriction in response to caffeine 5 mmol/L following treatment with vehicle (PSS) or ryanodine 20 μmol/L (n=6-7, N=5). Wilcoxon signed-rank test. **(D-F)** Relaxation of preconstricted arteries in response to sodium nitroprusside (SNP) following pretreatment with (D) ODQ 10 μmol/L (n=10, N=5), (E) Rp-8-CPT-cGMPs 1 mmol/L (n=10, N=5), or (F) KT5823 1 μmol/L (n=8, N=5). **(A, D-F)** Curves compared by 2-way RM ANOVA with Sidak's post-comparison tests. (A,B,C,E) Data are not normally distributed.

Supplemental Figure III

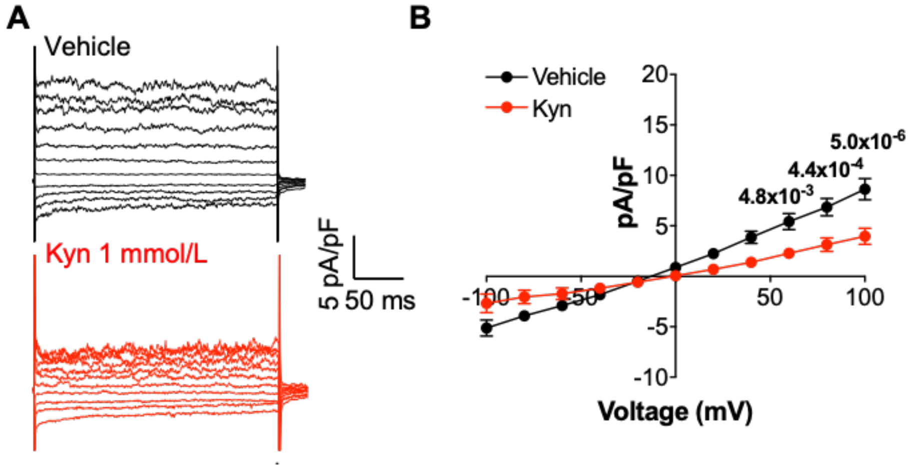

Supplemental Figure III: Kyn inhibits non-BK$_{Ca}$ outward current. (A) Representative traces of whole cell K^+ currents recorded in 20 mV steps (-100 to +100 mV) in isolated VSMCs in the presence of paxilline (1 μmol/L). VSMCs treated with vehicle (black) or Kyn (1 mmol/L; red). **(B)** Cumulative current-voltage characteristic curves (n=5-8, N=4). Curves compared by 2-way RM ANOVA with Sidak's multiple comparison tests.

Supplemental Figure IV

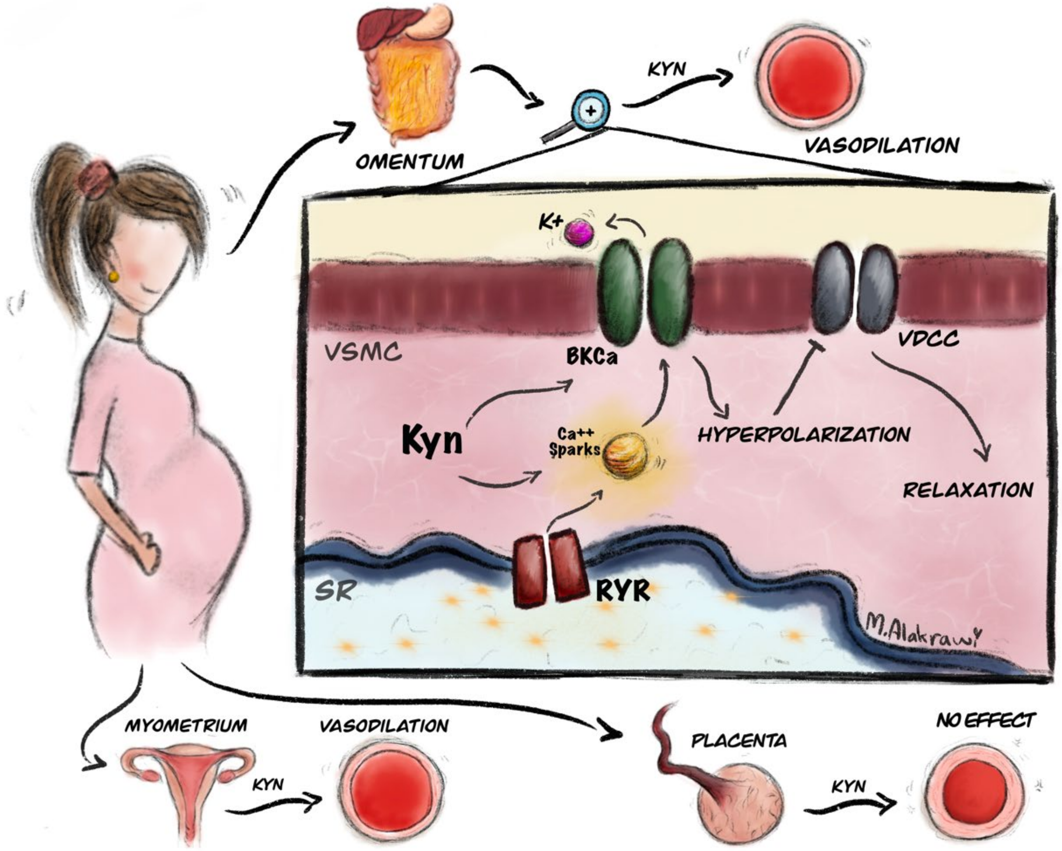

Supplemental Figure IV: A graphical abstract summarising the vasorelaxant effects of Kyn in arteries obtained from normotensive pregnant women. Kyn causes vasorelaxation in omental and myometrial arteries obtained from normotensive pregnant women, but does not cause relaxation in placental (chorionic plate) arteries. In omental arteries, Kyn causes relaxation via activation of BK_{Ca} channels. Kyn acts upon multiple parts of the Ca^{2+} spark-BK_{Ca} regulatory axis; Kyn has direct effects on BK_{Ca} channels and also causes an increase in the frequency of Ca^{2+} sparks.
