# Supplementary material for: Kynurenine Relaxes Arteries of Normotensive Women and Those With Preeclampsia
Source: Circ Res. 2021 Mar 3;128(11):1679–93. doi: 10.1161/CIRCRESAHA.120.317612 (PMC8154175; doi:10.1161/CIRCRESAHA.120.317612)
Supplement: Supplementary file 3 [file res-128-1679-s003.pdf]

Major Resources Table

In order to allow validation and replication of experiments, all essential research materials listed in the Methods should be included in the Major Resources Table below. Authors are encouraged to use public repositories for protocols, data, code, and other materials and provide persistent identifiers and/or links to repositories when available. Authors may add or delete rows as needed.

Animals (in vivo studies)

Species	Vendor or Source	Background Strain	Sex	Persistent ID / URL
N/A	N/A	N/A	N/A	N/A

Genetically Modified Animals

	Species	Vendor or Source	Background Strain	Other Information	Persistent ID / URL
N/A	N/A	N/A	N/A	N/A	N/A

Antibodies

Target antigen	Vendor or Source	Catalog #	Working concentration	Lot # (preferred but not required)	Persistent ID / URL
N/A	N/A	N/A	N/A	N/A	N/A

DNA/cDNA Clones

Clone Name	Sequence	Source / Repository	Persistent ID / URL
N/A	N/A	N/A	N/A

Cultured Cells

Name	Vendor or Source	Sex (F, M, or unknown)	Persistent ID / URL
N/A	N/A	N/A	N/A

Data & Code Availability

Description	Source / Repository	Persistent ID / URL
N/A	N/A	N/A

Other

Description	Source / Repository	Persistent ID / URL
1H-[1,2,4]oxadiazolo[4,3,-a]quinoxaline-1–one	Cayman Chemical	https://www.caymanchem.com/product/81410/odq<br>Product reference: 81410
4-Aminopyridine	Sigma -Aldrich	https://www.sigmaaldrich.com/catalog/product/aldrich/275875?lang=en&region=GB<br>Product reference: 275875
9-(Tetrahydro-2-furanyl)-9H-purin-6-amine (SQ 22,536)	Sigma -Aldrich	https://www.sigmaaldrich.com/catalog/substance/sq22536205221731831911?lang=en&region=GB<br>Product reference: S153
Amphotericin B	Sigma -Aldrich	https://www.sigmaaldrich.com/catalog/product/sigma/a4888?lang=en&region=GB<br>Product reference: A4888
Bradykinin acetate salt	Sigma -Aldrich	https://www.sigmaaldrich.com/catalog/product/sigma/b3259?lang=en&region=GB<br>Product reference: B3259
Caffeine	Sigma -Aldrich	https://www.labome.com/product/MilliporeSigma/27600.html

DOI [to be added]

		Product reference: 27600
Collagenase F	Sigma -Aldrich	https://www.sigmaaldrich.com/catalog/product/sigma/c7926?lang=en&region=GB<br>Product reference: C7926
Dithioerythritol	Sigma -Aldrich	https://www.sigmaaldrich.com/catalog/product/sial/d9680?lang=en&region=GB<br>Product reference: D9680
Fluo-4 AM	ThermoFisher Scientific	https://www.thermofisher.com/order/catalog/product/F14201#/F14201<br>Product reference: F14201
HEPES	Sigma -Aldrich	https://www.sigmaaldrich.com/catalog/product/sigma/h3375?lang=en&region=GB<br>Product reference:H3375
Iberiotoxin	Latoxan	https://www.latoxan.com/moleculars_product.php?id=1287&n=0<br>Product reference: L8211
KT5823	Sigma-Aldrich	https://www.sigmaaldrich.com/catalog/product/sigma/k1388?lang=en&region=GB<br>Product reference: K1388
Linopirdine	Sigma-Aldrich	https://www.sigmaaldrich.com/catalog/product/aldrich/l134?lang=en&region=GB<br>Product reference: L134
L-kynurenine	Sigma-Aldrich	https://www.sigmaaldrich.com/catalog/product/sigma/k8625?lang=en&region=GB<br>Product reference: K8625
NS11021	Sigma-Aldrich	https://www.sigmaaldrich.com/catalog/product/sigma/sml0622?lang=en&region=GB<br>Product reference: SML0622
Papain	Worthington biochemical	http://www.worthington-biochem.com/cls/cat.html<br>Product reference: LS004194
Paxilline	Sigma-Aldrich	https://www.sigmaaldrich.com/catalog/product/sigma/p2928?lang=en&region=GB<br>Product reference: P2928
Retigabine	Sigma-Aldrich	https://www.sigmaaldrich.com/catalog/product/sigma/sml0325?lang=en&region=GB<br>Product reference: SML0325
Rp-8-CPT-cGMPS	Sigma-Aldrich	https://www.sigmaaldrich.com/catalog/product/sigma/c240?lang=en&region=GB<br>Product reference: C240
Ryanodine	Tocris Bioscience	https://www.tocris.com/products/ryanodine_1329<br>Product reference: 1329
Sodium nitroprusside dihydrate	Sigma-Aldrich	https://www.sigmaaldrich.com/catalog/product/sigald/71778?lang=en&region=GB<br>Product reference: 71778
Tetraethylammonium chloride	Sigma-Aldrich	https://www.sigmaaldrich.com/catalog/product/sigma/t2265?lang=en&region=GB<br>Product reference: T2265
U46619	Sigma-Aldrich	https://www.sigmaaldrich.com/catalog/product/mm/538944?lang=en&region=GB<br>Product reference: 538944

DOI [to be added]
